# Issues with the Specificity of Immunological Reagents for NLRP3: Implications for Age-related Macular Degeneration

**DOI:** 10.1038/s41598-017-17634-1

**Published:** 2018-01-11

**Authors:** Cassandra Kosmidou, Nikolaos E. Efstathiou, Mien V. Hoang, Shoji Notomi, Eleni K. Konstantinou, Masayuki Hirano, Kosuke Takahashi, Daniel E. Maidana, Pavlina Tsoka, Lucy Young, Evangelos S. Gragoudas, Timothy W. Olsen, Yuki Morizane, Joan W. Miller, Demetrios G. Vavvas

**Affiliations:** 1 Department of Ophthalmology, Retina Service, Massachusetts Eye and Ear Infirmary, Harvard Medical School, Boston, Massachusetts, 02114 USA; 2 Department of Ophthalmology, Okayama University Graduate School of Medicine, Okayama, 700-8558 Japan; 3 Department of Ophthalmology, Mayo Clinic, 200 First Street SW, Rochester, MN 55905 USA

## Abstract

Contradictory data have been presented regarding the implication of the NACHT, LRR and PYD domains-containing protein 3 (NLRP3) inflammasome in age-related macular degeneration (AMD), the leading cause of vision loss in the Western world. Recognizing that antibody specificity may explain this discrepancy and in line with recent National Institutes of Health (NIH) guidelines requiring authentication of key biological resources, the specificity of anti-NLRP3 antibodies was assessed to elucidate whether non-immune RPE cells express NLRP3. Using validated resources, NLRP3 was not detected in human primary or human established RPE cell lines under multiple inflammasome-priming conditions, including purported NLRP3 stimuli in RPE such as *DICER1* deletion and *Alu* RNA transfection. Furthermore, NLRP3 was below detection limits in *ex vivo* macular RPE from AMD patients, as well as in human induced pluripotent stem cell (hiPSC)-derived RPE from patients with overactive NLRP3 syndrome (Chronic infantile neurologic cutaneous and articulate, CINCA syndrome). Evidence presented in this study provides new data regarding the interpretation of published results reporting NLRP3 expression and upregulation in RPE and addresses the role that this inflammasome plays in AMD pathogenesis.

## Introduction

Age-related macular degeneration (AMD) is the primary leading cause of vision loss in industrialized countries, with a worldwide prevalence of over 70 million patients and a projected increase of a quarter billion affected individuals by 2040^1,2^. The disease manifests in two major forms; the non-neovascular, non-exudative “dry” form affecting 85–90% of patients and the neovascular, exudative “wet” form affecting 10–15% of AMD patients. Improvements in understanding the implication of vascular endothelial growth factor (VEGF) in the pathogenesis of the wet form have led to effective therapies^3,4^, however the pathologic processes driving dry AMD remain elusive. Non-exudative AMD involves degeneration of the retinal pigment epithelium (RPE), a cell monolayer located between light-sensitive photoreceptor outer segments and the choroidal vasculature. RPE degeneration leads to photoreceptor dysfunction, death and vision loss. Although several culprits for dry AMD have been identified through epidemiological and genetic studies involving multiple biological pathways^5^, the degenerative processes of RPE and photoreceptors remain obscure. Altogether, this shortfall in understanding disease mechanisms, the lack of effective therapies and the disease’s soaring prevalence rates, highlights the significant unmet clinical need, as well as the necessity to address it.

Recent investigations in the field of AMD have drawn attention to the involvement of the NACHT, LRR and PYD domains-containing protein 3 (NLRP3, NALP3 or cryopyrin) inflammasome suggesting a key mediating role that drives RPE dysregulation and death. Inflammasomes are large intracellular multimeric protein complexes, which in response to infectious stimuli (pathogen-associated molecular patterns, PAMPs) and non-infectious danger signals induced by cellular stress and dying cells (danger-associated molecular patterns, DAMPs)^6–8^, lead to caspase-1 activation^9^ and subsequent release of two potent pro-inflammatory cytokines; mature interleukin (IL)-1β and IL-18. Inflammasome activation and production of mature IL-1β and IL-18 requires two signals; priming and activation. Priming involves NF-κB-mediated synthesis of the inactive precursors pro-IL-1β and pro-IL-18 and up-regulation of inflammasome components, including NLRP3. Following priming, activation is achieved through a second distinct signal, leading to NLRP3 oligomerization, recruitment of Apoptosis-Associated Speck-Like Protein Containing CARD (ASC) and procaspase-1, cleavage of procaspase-1 into active caspase-1, which in turn leads to cleavage and maturation of IL-1β and IL-18^10^. Ultimately, inflammasome activation most often leads to pyroptosis, a cell death pathway required to release these pro-inflammatory cytokines.

Inflammasomes are typically synthesized by immune cells. The notion that NLRP3 is involved in AMD was first described in 2012 when Doyle *et al*., suggested that components of sub-RPE drusen deposits from AMD patients could activate the NLRP3 inflammasome and caspase-1 in peripheral myeloid and mononuclear cells, leading to secretion of mature IL-1β and IL-18. IL-18 was found to be protective in a laser-induced choroidal neovascularization (CNV) animal model of neovascular AMD^11,12^. On the contrary, Tarallo *et al*., proposed that non-immune cells such as the RPE can generate NLRP3 and secrete damaging mature IL-18, in response to the accumulation of *Alu* RNA transposable elements, secondary to *DICER1* downregulation^13^. Subsequent studies also reported NLRP3 inflammasome activation in cultured RPE cell lines under various stimulations^14–20^. Hence, contradictory data have been presented regarding the implication of the NLRP3 inflammasome in AMD and on the proposed therapeutic strategies.

A critical component of experimental studies involves the quality, validity and reliability of resources used as tools to obtain data from which conclusions are drawn. Significant literature is devoted to the hurdle of published data irreproducibility^21–23^. Despite best efforts, most experimental findings contain errors^21^ and only 10–25% of key landmark pre-clinical studies have been replicated by independent groups^24,25^. Though there are many explanations, part of these disheartening results have been attributed to poorly described methodologies and ill-defined antibodies. More specifically, up to half of commercially available antibodies have been found to be unreliable^26,27^ and this reproducibility issue – or lack thereof – has been a recent matter of concern to the National Institutes of Health (NIH), which recently manifested: “*Research performed with unreliable or misidentified resources can negate years of hard work and eliminate any chance for a study to be reproduced or expanded upon. For this reason, it is imperative that researchers regularly authenticate key resources used in their research*”^28^. Thus, in line with NIH guidelines requiring the authentication of key biological and chemical resources, this study attempted to validate immunological reagents used in the study of the NLRP3 inflammasome, given the conflicting data published on AMD work and recognizing that antibody specificity may in part explain this discrepancy. A validated antibody was then utilized to elucidate whether NLRP3 is expressed in non-immune cells such as the RPE and if its expression is altered by known pathogenic stimuli that may play a role in AMD. Both established and primary human RPE cell lines under baseline or stimulated conditions as well as human RPE specimens of AMD patients and controls were examined for the presence of NLRP3. In summary, validated resources showed no NLRP3 expression in RPE in baseline, stimulated, healthy or diseased states.

## Results

### Validation of commercially available anti-NLRP3 antibodies by Western blotting

**Figure 1:**
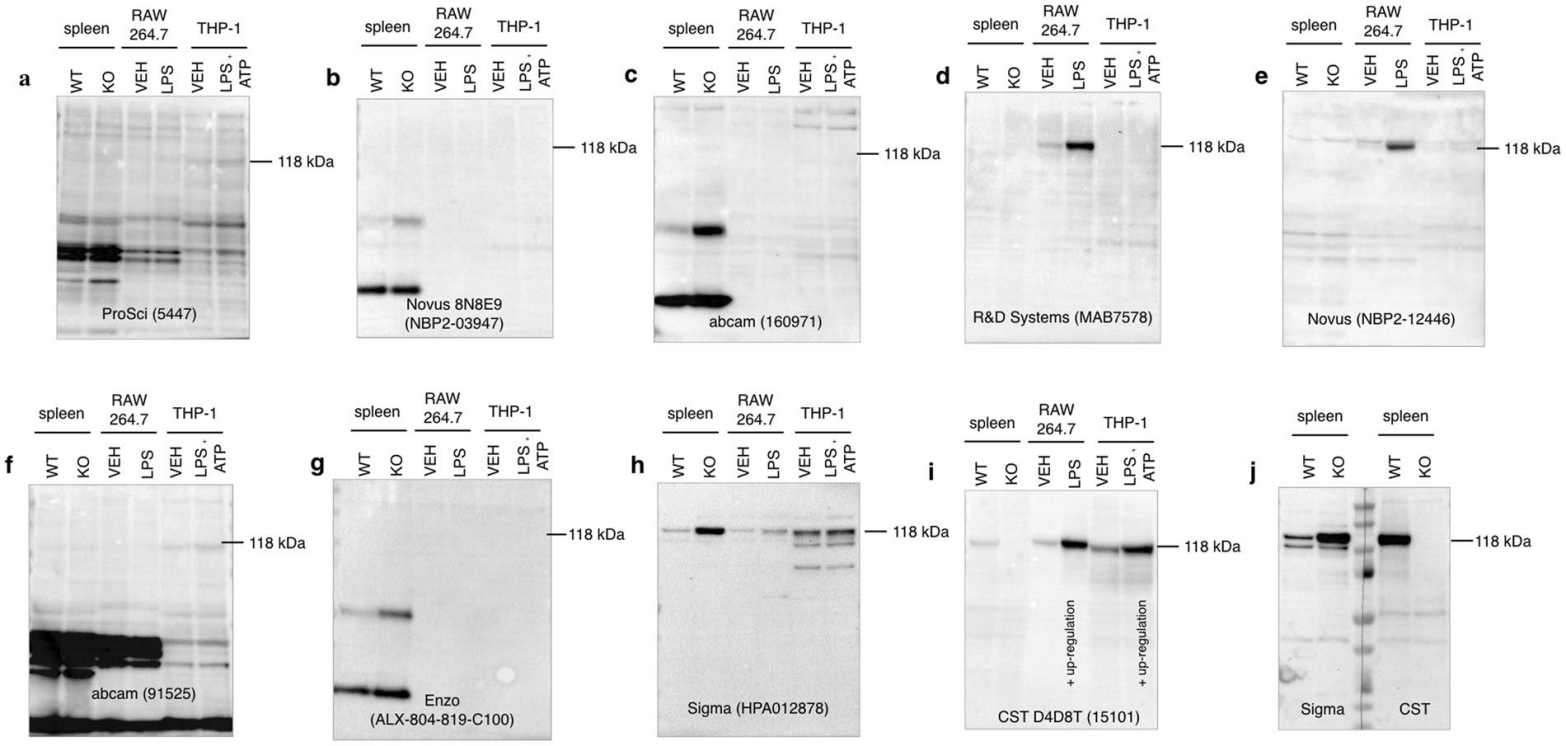
Validating the specificity of commercially available anti-NLRP3 antibodies by Western blotting. Nine commercially available anti-NLRP3 antibodies were tested in terms of their specificity against murine and human positive controls, including mouse spleen tissue, murine RAW 264.7 and human THP-1 macrophage cell lines. Antibody specificity was validated by testing protein expression in spleen tissue from *Nlrp3* knockout mice as a negative control. RAW 264.7 cells were primed with (LPS 10 ng/mL) for 6 hours and were compared to vehicle untreated cells. THP-1 macrophages were primed with LPS (10 μg/mL) plus ATP (5 mM) for 3 hours and compared to vehicle control THP-1. 50 μg of total protein were loaded on a gel and blotted with anti-NLRP3 antibodies with the expected molecular weight at ~118 kDa. The positive control panel was blotted with the following anti-NLRP3 antibodies. (**a**) ProSci, 5447, (**b**) Novus 8N8E9, NBP2-03947, (**c**) abcam, 160971, (**d**) R&D Systems, MAB7578, (**e**) Novus NBP2-12446, (**f**) abcam, 91525, (**g**) Enzo ALX-804-819-C100, (**h**) Sigma HPA012878 and (i) Cell Signaling Technologies (D4D8T, 15101). Blots were exposed for 10 minutes. CST (15101) shows a specific band at the expected molecular weight for all positive controls, which is absent in spleen from *Nlrp3* ^*−/−*^ sample. The response was amplified in stimulated RAW 264.7 and stimulated THP-1 cells compared to vehicle controls, demonstrating an upregulation of NLRP3 protein levels following inflammasome priming. In (**j**) duplicate samples of wild type and *Nlrp3* ^*−/−*^ spleens were run in parallel, membrane was cut to probe with CST (15101) or Sigma (HPA012878) antibodies to verify their differences. An informative list of the antibodies is shown in Supplementary Table S1. Each blot was repeated in three biologically independent replicates (*n* = 3) and equal protein loading was confirmed by Coomassie Blue staining (Supplementary Fig. S2). WT: wild type, KO: knockout, VEH: vehicle.

The specificity and sensitivity of commercially available anti-NLRP3 antibodies that have been previously used in AMD research (Supplementary Table S1)^11,13–15,17,19,20,29–39^ was assessed by examining their compliance to three validation criteria; Firstly, by means of a positive control, the signal of which had to be upregulated in the presence of an NLRP3-priming stimulus in immune cells. Secondly, the signal had to be abolished in tissue samples from *Nlrp*3 knockout mice and finally, the protein detected had to be of the correct molecular weight (~118 kDa Uniprot entry Q8R4B8 for mouse and Q96P20 for human). Spleen tissue and macrophages are known to express significant levels of NLRP3 protein^40^, hence mouse spleen, murine RAW 264.7 and human THP-1 macrophage cell lines were used as positive controls for antibody validation. Antibody specificity was assessed by comparing signals between spleen tissue from wild type (C57BL/6J) and *Nlrp3* knockout (B6.129S6-Nlrp3^tm1Bhk^/J) mice. Figure 1 illustrates that none of the eight antibodies used in published studies related to AMD met all three validation criteria in both mouse and human samples. The anti-NLRP3 antibody by R&D Systems (MAB7578) (Fig. 1d) detected bands at 118 kDa in both baseline and stimulated RAW 264.7 macrophages, although it did not detect endogenous NLRP3 from mouse spleen tissue possibly because it was not sensitive enough. Additionally, (MAB7578) did not detect NLRP3 in human basal or stimulated THP-1 macrophages, despite its purported cross-reactivity with human specimens. The Novus antibody (NBP2-12446) (Fig. 1e), detected a band of the appropriate molecular weight in baseline and stimulated RAW 264.7 macrophages, but also displayed additional non-specific bands close to the correct molecular weight, that were also present in knockout tissue. In addition, it displayed low sensitivity for mouse spleen, basal and stimulated human THP-1 cells. The rest of the antibodies (Fig. 1a,b,c,f,g) showed no specific signal in either tissue or cell lines. Interestingly, the Sigma antibody (HPA012878) (Fig. 1h,j) showed a band of appropriate size that instead of being absent, it was up-regulated in the *Nlrp3* knockout tissues. Furthermore, that same antibody could not detect upregulation of NLRP3 in stimulated immune cells. Although none of the previously used NLRP3 antibodies in AMD research met all validation criteria, a recently developed monoclonal anti-NLRP3 antibody (CST, D4D8T, 15101) (Fig. 1i,j) which has not been used in previously published AMD-related research, met all three criteria for specificity, sensitivity and signal induction. In summary, these data taken together suggest that none of the antibodies utilized in published work on NLRP3 inflammasome and AMD are reliable, since they are not specific for NLRP3.

### Sensitivity of validated anti-NLRP3 antibody (CST, 15101), Immunoprecipitation of NLRP3 in RPE and immune cells and NLRP3 protein sequencing analysis by mass spectrometry

**Figure 2:**
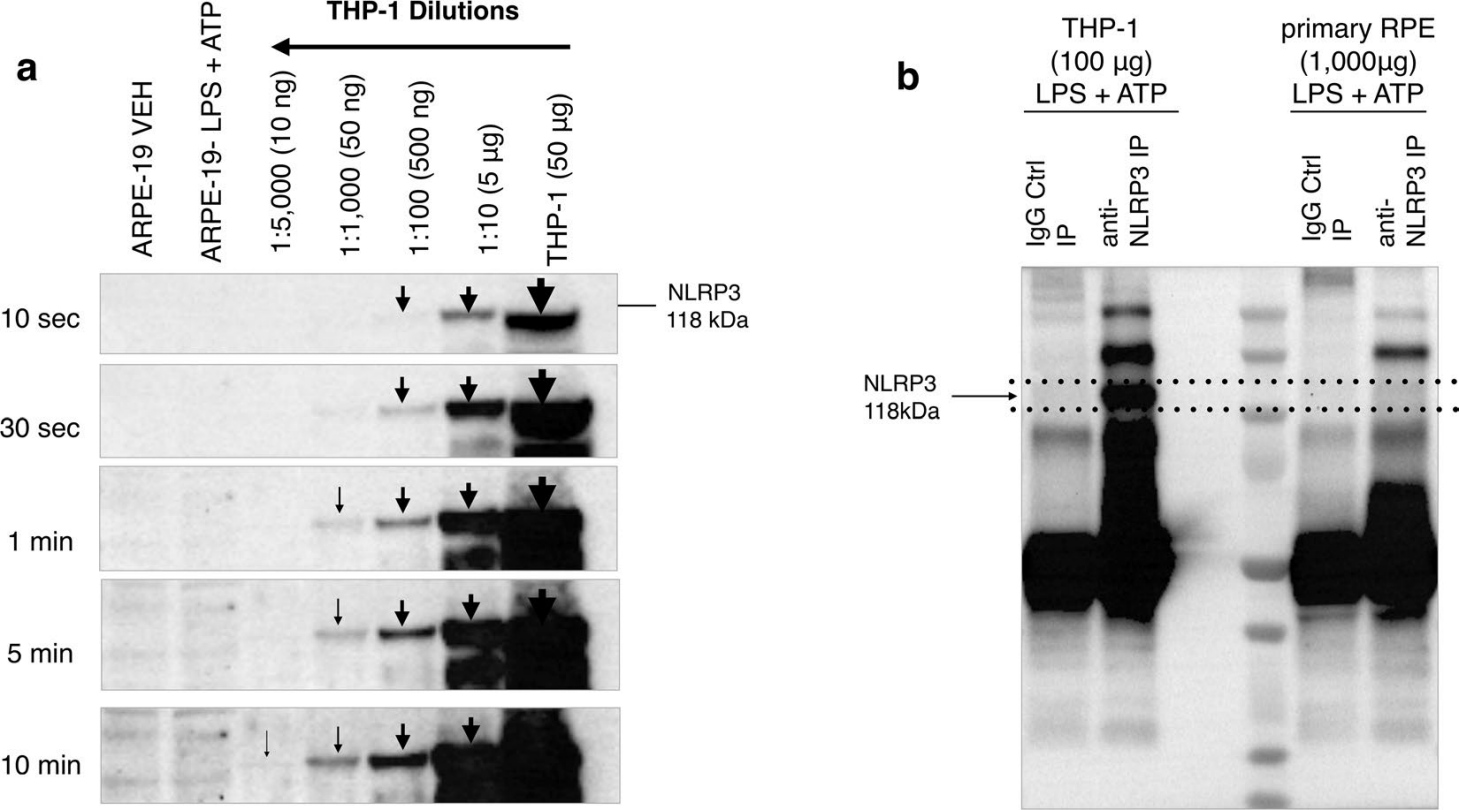
Sensitivity and immunoprecipitation assay for anti-NLRP3 antibody CST (D4D8T, 15101). (**a**) Serial dilutions of THP-1 cell lysate with starting total protein concentration of 50 μg (1:10, 1:100, 1:1000, 1:5000 in lysis buffer) demonstrate that the anti-NLRP3 antibody (CST D4D8T, 15101) was sensitive enough to detect NLRP3 from a protein sample diluted down to 10 ng of total THP-1 protein content. NLRP3 was not detectable in 25 μg protein samples of baseline or stimulated (LPS 10 μg/mL plus ATP 5 Mm, 24 hours) ARPE-19 cells at a 10-minute exposure time. In Fig. 2a the blot is presented at different exposure times (10 sec, 30 sec, 1 min, 5 min, 10 min). In addition, the blot is cropped, thus the full-length blot is presented in Supplementary Figure S7. (**b**) Protein A/G agarose beads coupled with anti-NLRP3 antibody (CST, 15101) were used to immunoprecipitate NLRP3. An isotype anti-Rabbit IgG antibody was used as a negative control instead of the NLRP3 antibody. NLRP3 protein was successfully immunoprecipitated in vehicle control THP-1 cell lysate but not in primary RPE cells stimulated with LPS (10 μg/mL) and ATP (5 mM) for 24 hours. Equal protein loading was confirmed by Coomassie Blue staining (Supplementary Figure S3).

Having determined the antibody’s (CST, 15101) specificity, its sensitivity in Western blots was further investigated by performing serial dilutions of the positive control (THP-1 50 μg 5 μg, 500 ng, 50 ng, and 10 ng of total protein). As seen in Fig. 2a, the antibody could detect NLRP3 from THP-1 lysates with total protein content of as little as 10 ng at an exposure time of 10 minutes. Using the validated and highly sensitive antibody, this study further aimed to address whether the non-immune RPE cell contains significant amounts of NLRP3. As seen in Fig. 2a the antibody detected NLRP3 in 10 ng of THP-1 total protein, yet it did not detect NLRP3 in 25 μg (25,000 ng) of stimulated ARPE-19 cells (LPS 10 μg/mL and ATP 5 mM) (Fig. 2a). Immunoprecipitation was performed to further expand the antibody’s sensitivity limits and to examine the hypothesis that NLRP3 may exist in very low quantities in the RPE. After establishing the antibody’s ability to efficiently immunoprecipitate almost the whole amount of total NLRP3 present in spleen or THP-1 cell lysate (Supplementary Figure S1), immunoprecipitation was performed in primary human fetal RPE (hfRPE) cell lysates under NLRP3-stimulated conditions (LPS 10 μg/mL plus ATP 5 mM). As seen in Fig. 2b NLRP3 was not detected even in 1 mg (1,000,000 ng) total protein of stimulated human primary fetal RPE (hfRPE). This points towards the unlikelihood of baseline or stimulated hfRPE cells containing significant levels of NLRP3.

Further to knockout validation and immunoprecipitation, protein sequence analysis of the immunoprecipitated band from a THP-1 sample was performed, to confirm its identity. As seen in Supplementary Figure S8 the protein was identified as NLRP3.

### No Expression of NLRP3 in established human RPE cell lines and primary human RPE cultures under basal or stimulated conditions

**Figure 3:**
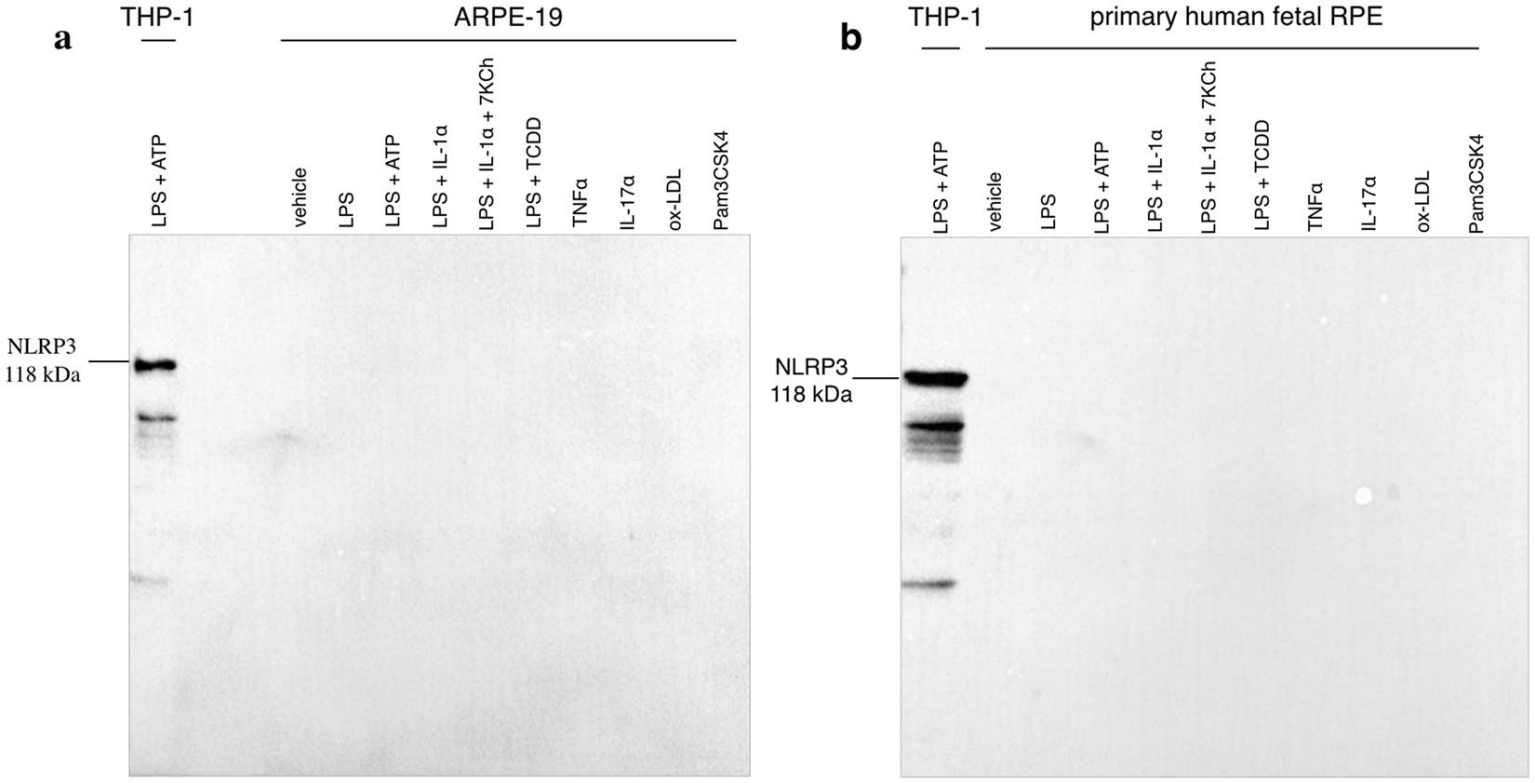
Absence of NLRP3 expression in human ARPE-19 cell line and primary human fetal RPE under basal and NLRP3-inducing stimuli. NLPR3 protein expression levels were assessed in human ARPE-19 (**a**) and primary human fetal RPE (**b**) cell lysates under basal conditions and NLRP3-priming stimuli. Both ARPE-19 and primary RPE cells were treated with LPS (10 μg/mL) alone, LPS (10 μg/mL) plus ATP (5 mM), LPS (10 μg/mL) plus TCDD (10 nM), LPS (20 μg/mL) plus IL-1α (25 ng/mL), LPS (20 μg/mL) plus IL-1α (25 ng/mL) plus 7-Ketocholesterol (10 μM), TNFα (10 ng/mL), IL-17α (100 ng/mL), Pam3CSK4 (300 ng/mL) for 24 hours and oxidized-LDL (500 μg/mL) for 48 hours. NLRP3 expression levels in ARPE-19 and primary RPE cells were compared to stimulated human THP-1 cell lysate LPS (10 μg/mL) plus ATP (5 mM) for 3 hours. The membrane was blotted with anti-NLRP3 antibody (CST, 15101) and no apparent bands were present to indicate NLRP3 expression in either cell line or primary cells at a 10-minute exposure time. Each blot was repeated in three biologically independent replicates (*n* = 3) and equal protein loading was confirmed by Coomassie Blue staining (Supplementary Figure S4).

Since several other stimuli reportedly stimulate NLRP3 inflammasome in RPE cells, NLRP3 levels were investigated in both primary (hfRPE) and established human RPE cell lines (ARPE-19) under basal levels and under reported inflammasome-stimulating conditions; (LPS 10 μg/mL alone, LPS 10 μg/mL plus ATP 5 mM, LPS 10 μg/mL plus TCDD 10 nM, LPS 20 μg/mL plus IL-1α 25 ng/mL, LPS 20 μg/mL plus IL-1α 25 ng/mL plus 7-Ketocholesterol 10 μM, TNFα 10 ng/mL, IL-17α 100 ng/mL, Pam3CSK4 300 ng/mL for 24 hours and oxidized-LDL 500 μg/mL for 48 hours). As seen in Fig. 3a and b, NLPR3 was not detected in either ARPE-19 or primary hfRPE cells under any conditions at a 10-minute exposure time. Furthermore, none of the inflammasome-activating stimuli caused a statistically significant increase in *NLRP3* mRNA and transcript variant levels in ARPE-19 cells under any of the stimulations tested (Table 1). Collectively, these data indicate that neither *NLRP3* transcripts nor protein levels are upregulated in human RPE cells following treatment with previously described NLRP3 stimuli.

**Table 1:** Relative *NLRP3* mRNA expression in baseline and stimulated THP-1 and ARPE-19 cells.

	Vehicle	LPS	LPS + ATP	LPS + IL1α	LPS + IL1α + 7KCh	TNFα	Pam3CSK4	ox-LDL	LPS + TCDD	IL-17	THP-1 Veh	THP-1 (LPS + ATP)
Mean fold change	1.0	0.9	1.0	1.9	0.8	0.9	0.5	0.4	0.4	0.5	719	1019
SEM	0.0	0.3	0.4	1.0	0.7	0.6	0.3	0.3	0.2	0.2	25.3	62
Avg fold change exp #1	1.0	0.7	1.7	0.8	0.0	0.2	0.1	0.2	0.0	0.7		
Avg fold change exp #2	1.0	0.5	0.4	1.0	0.3	0.3	0.5	0.1	0.4	0.1	744.8	1081.1
Avg fold change exp #3	1.0	1.5	0.8	3.9	2.1	2.1	0.9	1.0	0.6	0.7	694.3	957.2
	**Ct Values**											
Experiment 1 NLRP3	35.9	36.9	36.0	41.0	39.8	41.0	41.0	37.1	41.0	36.8		
	41.0	35.2	36.9	36.2	38.2	41.0	36.7	41.0	41.0	37.1		
	37.0	37.2	34.8	35.8	39.8	35.8	41.0	41.0	38.1	36.7		
Exp.1 reference RNA	26.4	24.7	25.8	26.0	23.0	25.3	24.1	26.0		24.6		
	26.3	24.2	25.0	25.8	22.4	25.0	23.9	25.6	23.4	24.6		
		23.7	24.1	25.7	22.0	25.0	23.7	25.3	23.7	24.8		
Experiment 2 NLRP3	36.5	37.1	37.3	41.0	38.6	41.0	37.1	41.0	41.0	41.0	30.9	30.6
	36.9	41.0	37.0	37.0	39.4	36.8	41.0	41.0	41.0	41.0	31.2	31.1
	37.1	36.7	41.0	36.3	38.7	41.0	37.1	41.0	41.0	41.0	30.2	30.2
Exp.2 reference RNA	18.3	18.7	18.6	19.5	18.6	19.4	18.9	19.2	21.0	18.8	21.4	21.9
	18.2	18.6	18.6	19.5	19.0	19.4	19.0	19.1	21.0	18.9	22.0	22.3
	18.2	18.6	18.6	19.5	18.7	19.4		19.1	21.1	18.8	21.4	21.7
Experiment 3 NLRP3	39.0	41.0	39.8	37.9	37.5	37.6	39.9	38.9	41.0	39.9	28.5	28.2
	41.0	37.3	41.0	38.6	41.0	38.9	41.0	41.0	41.0	41.0	28.4	28.1
	39.1	38.1	41.0	38.8	37.3	39.0	41.0	39.1	38.9	39.9	28.3	27.9
Exp.3 reference RNA	20.9	20.6	21.9	21.6	20.9	20.9	21.8	21.0	21.0	21.9	19.3	19.2
	21.0	21.0	21.1	21.8	21.0	21.0	21.6	20.8	20.8	20.5	19.0	19.2
	20.9	20.3	21.7	21.4	20.9	20.5	21.9	20.9	20.8	20.8	19.3	19.3

Expression levels of NLRP3 mRNA measured in baseline and stimulated THP-1 and ARPE-19 cells were analyzed by RT-PCR. THP-1 cells were primed with LPS (10 μg/mL) plus ATP (5 mM) for 4 hours. ARPE-19 cells were stimulated with NLRP3-priming stimuli including LPS (10 μg/mL) alone, LPS (10 μg/mL) plus ATP (5 mM), LPS (10 μg/mL) plus TCDD (10 nM), LPS (20 μg/mL) plus IL-1α (25 ng/mL), LPS (20 μg/mL) plus IL-1α (25 ng/mL) plus 7-Ketocholesterol (10 μM), TNFα (10 ng/mL), IL-17α (100 ng/mL), Pam3CSK4 (300 ng/mL) for 24 hours and oxidized-LDL (500 μg/mL) for 48 hours. In ARPE-19 samples, expression levels are shown as fold changes in mRNA, relative to ARPE-19 vehicle from three biologically independent experiments (n = 3), that were performed in triplicates. Values expressed as mean fold changes in expression; 2^−ΔΔCt^ (2^−^(ΔCt - ΔCtvehicle)) ± standard error of the mean (SEM) and threshold cycle (Ct) values.

### Absence of NLRP3 expression in RPE primary or established cell lines after DICER1 deletion and Alu RNA transfection

**Figure 4:**
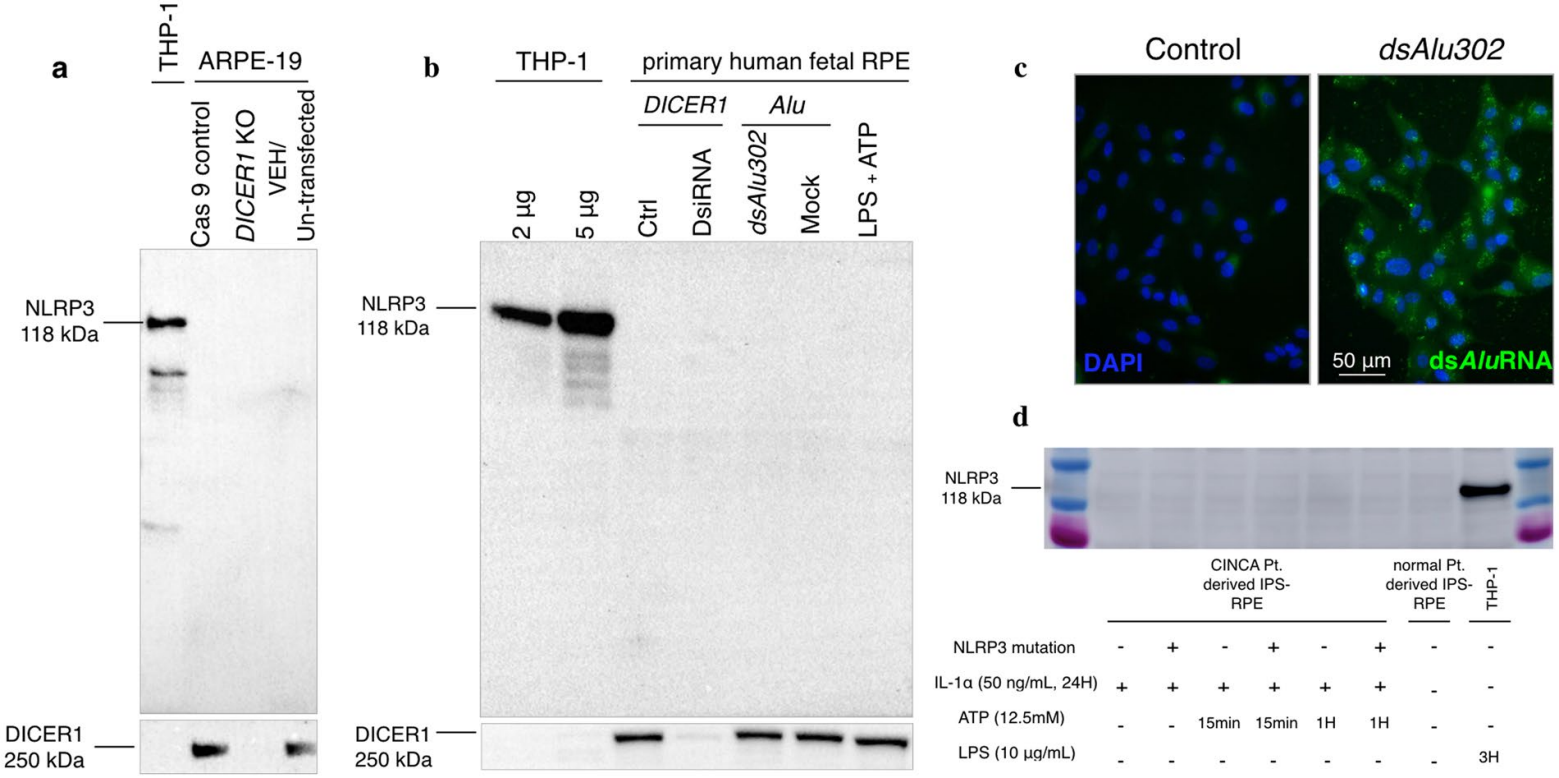
Absence of NLRP3 expression in human ARPE-19 cell line and primary human fetal RPE following *DICER1* deletion or downregulation and ds*Alu* RNA transfection. Absence of NLRP3 expression in iPSC-RPE from CINCA patients. NLPR3 protein expression levels were assessed in human ARPE-19 cells following *DICER1* knockout via CRISPR/Cas9 (**a**) and *DICER1* knockdown via DsiRNA in primary human fetal RPE (**b**). Analysis was performed by Western blotting, using the CST NLRP3 antibody (CST, 15101). No observed changes in NLRP3 expression were seen following *DICER1* knockout or knockdown compared to Cas9 and mock transfection controls. Transfection of stimulated primary human fetal RPE cells (LPS 10 μg/mL plus ATP 5 mM for 24 hours) with ds*Alu302* RNA did not cause any changes in NLRP3 levels compared to mock transfection control (**b**). Signals were compared to stimulated THP-1 cells LPS (10 μg/mL) plus ATP (5 mM) for 3 hours and images were taken after a 10-minute exposure. Successful transfection of primary RPE cells with ds*Alu302* was validated by immunofluorescence compared to control cells (blue: DAPI, green: *dsAlu302* RNA) (**c**). Figure 4d illustrates the absence of NLRP3 protein in differentiated RPE generated from iPSCs from CINCA patients, with and without the *NLRP3* mutation. NLRP3 was absent both in baseline and stimulated IL-1α (50 ng/mL, 24 hours), ATP (12.5 mM) CINCA-derived iPSC-RPE cells. Equal Protein loading was confirmed by Coomassie Blue staining (Supplementary Figure S5).

Since DICER1 deficiency and *Alu* RNA accumulation have been suggested as the original stimuli for NLRP3 inflammasome activation in RPE from AMD patients, *DICER1* knockout was performed via CRISPR/Cas9 genome editing in ARPE-19 cells. As observed in Fig. 4a, *DICER1* deletion did not lead to any detectable NLRP3 induction in ARPE-19 cells. Similarly, primary hfRPE cells with efficient *DICER1* knockdown using DsiRNA, or primary hfRPE cells with *dsAlu302* RNA transfection failed to show detectable NLRP3 protein (Fig. 4b). Successful DICER Knockout was confirmed by Western blotting and successful transfection of primary hfRPE cells with *dsAlu302* RNA was validated by immunofluorescence (Fig. 4c). Concluding, DICER1 ablation and ds*Alu* RNA induction did not induce NLRP3 in human primary or established RPE cells.

### Absence of NLRP3 expression in iPSC-derived RPE from CINCA patients and in macular RPE from AMD patients

NLRP3 levels were investigated in RPE from patients with an activating mutation in the gene that encodes for NLRP3 (*CIAS1)*. This results in a severe congenital inflammatory disease called chronic infantile neurologic cutaneous and articulate (CINCA) syndrome^41^ due to chronic *NLRP3* activation. Hence, hiPSC-derived RPE from CINCA patients were tested for NLRP3 expression. As seen in Fig. 4d, no detectable NLRP3 was observed in hiPSC-RPE from patients with or lacking the *NLRP3* mutation even following stimulation with IL-1α (50 ng/mL) and ATP (12.5 mM).

**Figure 5:**
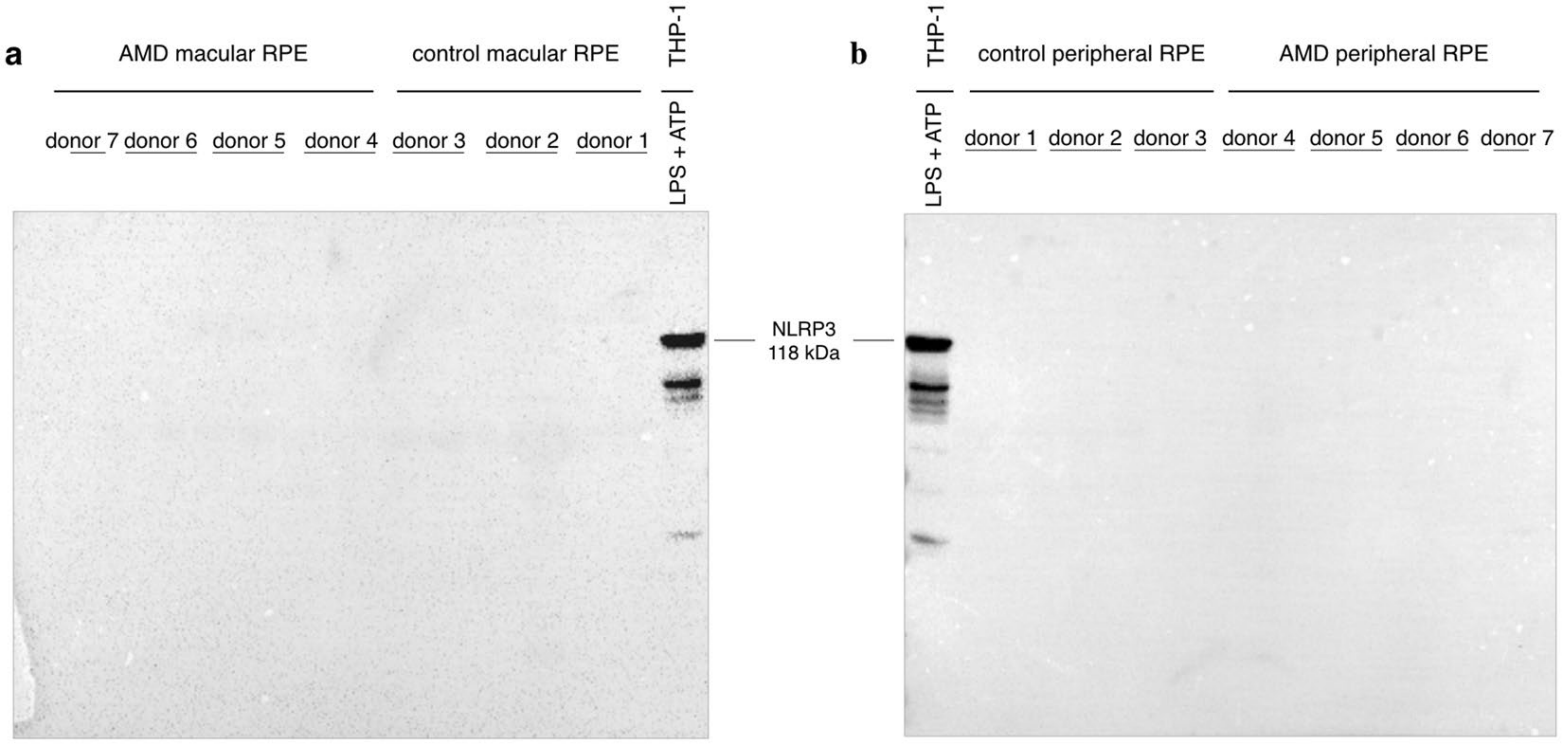
Western blot analysis of NLRP3 in human macular and peripheral RPE from AMD donors and age-matched controls. Human macular and peripheral RPE lysates were collected from four AMD donors (7 globes) and three age-matched non-AMD controls (6 globes). NLRP3 levels in macular and peripheral RPE samples were examined by Western blotting using anti-NLRP3 antibody (CST, 15101). Positive control used included primed THP-1 macrophages (LPS 10 μg/mL and ATP 5 mM, 3 hours). Blots were exposed for 10 minutes and the absence of specific bands in either AMD or age-matched control RPE samples indicates that NLRP3 is unlikely to be expressed in human RPE cells. Protein loading was confirmed by Coomassie Blue staining (Supplementary Figure S6).

Finally, NLRP3 protein expression was investigated in human macular and peripheral RPE cells from dry AMD donors (7 globes) and 4 age-matched non-AMD healthy controls (6 globes) by Western blotting. As illustrated in Fig. 5, NLRP3 protein was below detection limits in RPE lysates from either dry AMD or age-matched healthy controls at 10-minute exposure times. To maximize NLRP3 detection signals, equal protein loading was not performed, which would have limited the assay to the most dilute sample. Instead, maximum amount of protein from each sample was loaded (Supplementary Figure S6).

## Discussion

This study demonstrates the issues with specificity of key reagents used to study NLRP3 in RPE in AMD, which questions current interpretation of results reporting NLRP3 expression and upregulation in AMD. It highlights the importance of the implementation of the new NIH requirements for proper authentication and validation of key biological resources^28^. As seen in Fig. 1, only one antibody (CST D4D8T, 15101) was rendered specific and sensitive for NLRP3 in both murine and human samples and two other antibodies were able to only recognize NLRP3 highly expressing RAW cells but did not exhibit high enough sensitivity for spleen tissue or human THP-1 cells. Hence, out of the antibodies tested in this study, only the CST antibody can be utilized to safely address a key biological question of major clinical importance in the field of AMD. Does a non-immune cell such as the RPE contain significant amounts of NLRP3 under basal or stimulated conditions that could contribute to AMD pathogenesis? Several lines of evidence presented in this study show that this is unlikely to be the case.

The majority of prior published reports on NLRP3 and RPE fail to show clear NLRP3 expression on the protein level (Western blotting) using the appropriate positive and negative controls^13,15,17,31,34^, or they do not include adequate protein expression analysis. Using a validated and authenticated antibody for NLRP3, protein expression was not detected in the well-established human cell line ARPE-19 under baseline or stimulated conditions (Fig. 3). Although this human RPE cell line has previously been used to study NLRP3 with these stimuli^14,15,19,30,32,42–44^, it is argued that ARPE-19 cells are unlike true RPE cells since they exhibit fibroblastic characteristics^45^. Thus, it is thought to be more appropriate to use primary RPE cells in addition to established transformed cell lines. In agreement with results from ARPE-19 cells, NLRP3 protein was not detected in primary hfRPE under purported NLRP3 inflammasome-stimulating conditions (Fig. 3). The first report to propose that RPE cells express NLRP3, suggested that this was due to DICER1 downregulation, leading to *Alu* RNA accumulation and NLRP3 upregulation^13,31^. However, as seen in Fig. 4, despite complete loss of *DICER1* in ARPE-19, significant loss of *DICER1* in hfRPE, or ds*Alu* RNA transfection in both RPE lines, no detectable NLRP3 was observed. This study provides evidence that complete *DICER1* ablation leaves NLRP3 levels unaffected in RPE cells below the detection limit of this very sensitive antibody. However, since culturing cells alters their physiology, in addition to using human RPE cell lines and primary cultures, human macular and peripheral RPE lysates were collected from dry AMD donors and age-matched non-AMD controls. As illustrated in Fig. 5, NLRP3 protein was below detection limits in RPE lysates from both AMD and age-matched control donors.

CINCA syndrome is a severe congenital inflammatory disease, which, in 50% of patients is caused by activating mutations in *CIAS1*, a gene encoding NLRP3 (also known as cryopyrin)^41^. This leads to chronic activation of *NLRP3*, hence hiPSC-derived RPE from CINCA patients were tested for NLRP3 expression. No detectable NLRP3 was observed in hiPSC-RPE from patients with the *CIAS1* mutation under NLRP3-priming stimuli (Fig. 4d). It is interesting to note that although these patients reportedly have elevated systemic IL-18 and IL-1β^46^ and can be affected by intraocular inflammation^47^, macular degeneration-like phenotype has not been described thus far^48^.

These data taken together strongly suggest that if NLRP3 is expressed in the RPE, it could be present in very low levels that fall below the detection limit of this very sensitive antibody. The antibody’s sensitivity was determined to be quite high, detecting NLRP3 from a *bona fide* NLRP3-expressing cell (THP-1) with total protein extract of only 10 ng. Nonetheless, this antibody did not detect NLRP3 in 25 μg of stimulated ARPE-19 extract (Fig. 2a). Thus, it can be deduced that if RPE cells express any NLRP3, expression levels are well below 1/1000^th^ of what a *bona fide* NLRP3-expressing cell contains. To further increase the sensitivity of our detection and to examine whether higher amounts of RPE extract contain detectable NLRP3, immunoprecipitation was performed. Hence, NLRP3 levels were measured from RPE lysates containing a total of 1,000 μg of protein which is much higher than the usual 25–50 μg of protein per sample loaded in a gel for Western blotting. After establishing that NLRP3 can be successfully immunoprecipitated in THP-1 immune cells (Supplementary Fig. S1), NLRP3 was still below detection limits in stimulated hfRPE cell lysates (Fig. 2b). In summary, the protein analysis presented in this study, revealed that NLRP3 could be detected in as little as 10 ng of total protein extract from THP-1 cells and was below detection limits from 1 million ng of stimulated human primary RPE extract. It is thus safe to conclude that no appreciable NLRP3 is present in the RPE even following a strong inflammasome stimulation.

Several studies tried to circumvent this lack of evidence of NLRP3 expression on the protein level by measuring *NLRP3* mRNA levels in RPE cells. These studies revealed a 1.4x to 2x fold induction of *NLRP3* in stimulated RPE cells compared to baseline levels^16,19,20,30^. However, none of these studies reported the threshold cycle (Ct) at which *NLRP3* mRNA was detected since data are presented as relative mRNA levels. Our RT-PCR analysis failed to detect any significant *NLRP3* mRNA induction in stimulated ARPE-19 samples compared to untreated vehicle ARPE-19 cells. The amplification signals–if any–were detected close to or exceeding 36 out of 40 cycles of PCR. A single experimental run using digital PCR to measure exact *NLRP3* mRNA copy numbers per stimulated ARPE-19 cell, revealed that the RT-PCR signals from RPE cells may represent less than 1 copy of mRNA per 50 cells further suggesting that the RPE is unlikely to contain significant levels of *NLRP3* mRNA.

In summary, the data presented in this study suggest the existence of several non-specific commercially available anti-NLRP3 antibodies that question current interpretation of results reporting NLRP3 expression and upregulation in the RPE of AMD patients. Our data suggest caution and question the interpretation that NLRP3 inflammasome is a driving force behind RPE dysfunction in non-exudative (dry) AMD. This study argues that RPE may not contain meaningful amounts of NLRP3 to contribute to diseased states. Furthermore, the data presented suggest that if NLRP3 is implicated in AMD, it is more likely to be related to immune cells, either resident or infiltrating^11,12,49–52^. Thus, further evidence is required to characterize the presence and, source and activation of proinflammatory cytokines (IL-18) in AMD. Finally, this work highlights the need for immunological reagent validation and authentication by using the appropriate positive and negative knockout controls, as well as guarded interpretation of experiments that are not rigorously controlled.

## Methods

### Reagents

A list of antibodies used is presented in Supplementary Table S1. LPS-EB Ultrapure (*E. coli* 0111: B4) and the TLR1/2 heterodimer receptor agonist Pam3CSK4 (tlrl-pms) were purchased from InvivoGen (San Diego, CA). ATP (A2383), Tetrachlorodibenzo*-p-*dioxin (TCDD, 48599), IL-17α (H7791) and IL-1α (I2778) were purchased from Sigma (St. Louis, MO). 7-Ketocholesterol (C6970-000) was obtained from Steraloids (Newport, RI), Tumor Necrosis factor α (TNFα, 210-TA) was acquired from R&D Systems (Minneapolis, MN) and oxidized low density lipoprotein (ox-LDL, BT-910) was purchased from Alfa Aesar (Tewksbury, MA).

### Animals

Animal procedures conformed to the guidelines proposed in the ARVO Statement for the Use of Animals in Ophthalmic and Visual Research and were approved by the Animal Care Committee of the Massachusetts Eye and Ear Infirmary. Mice were kept in 12-hour light/dark cycles and fed on standard lab diet with free access to water. Eight-week-old male C57BL/6 J (Stock No.000664) and *Nlrp3* ^*−/−*^(B6.129S6-Nlrp3^tm1Bhk^/J, Stock No. 021302) mice were purchased from Jackson Laboratories. The entire coding region of *Nlrp3* in *Nlrp3* ^*−/−*^ mice is replaced with a neomycin-resistant targeting vector leading to no production of NLRP3 protein^53^. Mice were anesthetized by Tribromoethanol injection (250 mg/kg, intraperitoneal i.p.) and sacrificed by cervical dislocation. Spleen tissue and eyes were dissected within one minute post-sacrifice.

### Cell culture procedures

ARPE-19 (CRL-2302) were purchased form ATCC (Manassas, VA) and primary human fetal RPE (hfRPE) cells (H-RPE, Lonza 00194987) were purchased from Lonza (Basel, Switzerland) and were preserved in a cell culture incubator set at 5% CO_2_, 37 °C. ARPE-19 cells were cultured in DMEM high-glucose medium containing 100 U/ml penicillin, 100 μg/ml streptomycin, 10 mM nicotinamide, 2 mM sodium pyruvate and supplemented with 5% heat inactivated Fetal Bovine Serum (FBS). Approximately 20-week old primary fetal RPE cells were seeded at passage 1 in RtEGM^TM^ Retinal Pigment Epithelial Cell Basal Medium (Lonza, 195407 2% FBS). 24 hours following seeding, hfRPE cells were cultured with RtEGM^TM^ without FBS. Population doubling occurred every 3–4 days and experiments were performed at passage 3–5. ARPE-19 and primary fetal hfRPE cells were treated with LPS (10 μg/mL) alone, LPS (10 μg/mL) plus ATP (5 mM), LPS (10 μg/mL) plus TCDD (10 nM), LPS (20 μg/mL) plus IL-1α (25 ng/mL), LPS (20 μg/mL) plus IL-1α (25 ng/mL) plus 7-Ketocholesterol (10 mM), TNFα (10 ng/mL), IL-17α (100 ng/mL), Pam3CSK4 (300 ng/mL) for 24 hours and oxidized-LDL (500 μg/mL) for 48 hours. THP-1 human monocytes (ATCC, TIB-202) were cultured in RPMI-1640 medium (ThermoFisher, 11875093) containing antibiotics and 10% FBS. THP-1 cells (passage 6) were primed with LPS (10 μg/mL) plus ATP (5 mM) for 3 hours. RAW-264.7 macrophages (ATCC, TIB-71, passage 6) were cultured in DMEM (ATCC, 30-2002) with antibiotics and 10% FBS and were primed with LPS (10 ng/mL) for 6 hours. 24 hours prior to treatment, cell cycles were synchronized by serum starvation and treatments were performed in serum-free media. The above time-points and reagent concentrations were selected to replicate previously published work on upregulation of NLRP3 in ARPE-19 and hfRPE cells under those conditions.

#### *DICER1* knockout ARPE-19 by CRISPR/Cas9

Three CRISPR targeting sequences were designed based on the Optimized CRISPR Design web tool (http://crispr.mit.edu/), and listed on Supplementary Table S2. Oligos were cloned into the pSpCas9 (BB)-2A-Puro (PX459) (Addgene, plasmid #62988) following the genome engineering protocol by Ran *et al*.^54^ ARPE-19 were seeded at 75% of confluence and transfected with three plasmids containing a single guideRNA. Transfection was achieved by Lipofectamine3000 Reagent (Invitrogen, L3000008) according to the manufacturer’s protocol. Cells were treated in media containing 3 μg/ml puromycin (Santa Cruz, sc-108071B) for three days and successful *DICER1* deletion was confirmed by Western blotting.

#### *DICER1* knockdown with DsiRNA and *dsAlu* RNA transfection in human RPE

DsiRNA sequences were designed using IDT integrated DNA technology software (www.idtdna.com), ID number (hs.RiDICER1.13.2) which were located in exon 23. Double stranded RNA *Alu* (ds*Alu*) was made as previously described^13^. Briefly, ds*Alu* was synthesized and purified for RPE transfection from a PCR fragment of *Alu* cDNA containing T7 and T3 promoters at both ends. DsiRNA generation and ds*Alu* transfection into RPE were achieved by using a TransIT-mRNA Transfection Kit (Mirus Bio, MIR2252) with various combinations of transfecting component ratios, including 100 μl of OptiMEM mixed with 2 μl of TransIT-mRNA Reagent, 2 μl Boost and 2 μg of total DsiRNA or ds*Alu302*. Following a 3-minute incubation, the mixture was added to 12-well plates containing 1 ml medium with RPE growing at 95% confluence. Transfection efficiency was confirmed after 24 hours with TYE-563/DsiRNA or immunofluorescence for dsRNA (SCICIONS, 10010200).

#### Human induced Pluripotent Stem Cell (hiPSC) derived cell line culture and differentiation into RPE

hiPSC cell lines (HPS0119 and HPS0120) were generated from skin cells from CINCA-syndrome patient carrying an *NLRP3* mutation as somatic mosaicism. HPS0119 is a mutant hiPSC clone and HPS0120 is a wild-type iPSC clone from same patient. Control hiPSC line (454E2) was generated from dental pulp cells from healthy donors (RIKEN BioResource Center). hiPSCs were maintained and differentiated as previously described^55^. Undifferentiated hiPSCs were maintained on mouse embryo fibroblast (MEF) feeder cells in Primate ES medium (ReproCELL, Japan, Kanagawa) supplemented with 5 ng/ml basic fibroblast growth factor (βFGF, ReproCELL) and fresh medium was added daily. hiPSCs were passaged in small clumps after treatment with dissociation solution for iPSCs and replaced onto MEFs every 7 days. To differentiate into RPE, hiPSCs were cultured on gelatin-coated dishes in differentiation medium (GMEM, GIBCO, Grand Island, NY, USA) supplemented with 1 mM sodium pyruvate, 0.1 mM non-essential amino acids, 0.1 mM 2-mercaptoethanol and 20% KnockOut^TM^ Serum Replacement (KSR, GIBCO) for 4 days, GMEM and 15% KSR for 6 days, and GMEM and 10% KSR for 18days. At 4 weeks, pigmented cells with a typical RPE cobblestone appearance appeared focally and the differentiation medium was switched to SFRM containing DMEM/F12 [7:3] supplemented with B27, GIBCO, 2 mM L-glutamine for 7 days. Pure populations of pigmented cells were obtained by transferring pigmented colonies to dishes in SFRM supplemented with 10 ng/ml βFGF and 0.5 μM SB431542 (Sigma, S4317) and differentiation medium was changed every three days. CINCA-syndrome patient-derived hiPSC-RPE (passage 4) were stimulated with IL-1α (50 ng/mL) for 24 hours and ATP (12.5 mM) for 15 minutes or 1 hour. Using the anti-NLRP3 antibody (CST, 15101) NLRP3 protein expression was examined by Western blotting.

### Western Blotting

Following dissection, animal tissues were immediately immersed in ice-cold lysis buffer and were homogenized (polytron), while adherent cells were scraped off with lysis buffer; all samples were sonicated at 20% amplitude (10 seconds). Protein was extracted in lysis buffer containing 20 mM NaHEPES, 20 mM KCl, 20 mM NaF, 20 mM glycerophosphate, 2 mM sodium pyrophosphate, 2.5 mM EGTA, 2.5 mM EDTA, 1%Triton x-100, 0.1% 2-Mercaptoethanol and protease inhibitor cocktail (Roche, 11836170001). Lysates were cleared (17,000 × g, 20 minutes, 4 °C) and total sample protein quantification was measured by a Bradford assay (ThermoFisher, 23236). Samples were reduced with 2.5% 2-Mercaptoethanol and SDS sample buffer, loaded on 4–12% Bis-Tris Polyacrylamide gels and proteins were separated by electrophoresis at 200 V for 50 minutes. Proteins were transferred on a 0.45 μm PVDF membrane at 130 V for 1,5 hours. Successful transfer and equal protein loading were confirmed by Brilliant blue staining (Sigma, B2025) (0.1% Brilliant blue in 50% methanol, 10% acetic acid, 40% dH2O). Membranes were de-stained by washing with 70% methanol 10% acetic acid, 20% H_2_O, 0.1% 10 N NaOH. Membranes were incubated with blocking solution 5% milk in TBS with 1% Triton X-100 (TBST) for 30 minutes in room temperature, primary antibodies (Supplementary Table S1) were diluted in blocking solution and incubated at 37 °C for 30 minutes. Membranes were washed in TBST 3 times for 3 minutes each and incubated with secondary HRP-conjugated antibody (Cell Signaling, 1: 20,000 in blocking solution) at 37 °C for 20 minutes. Membranes were washed for 3 minutes 3 times and positive signals were detected by chemiluminescence ECL Select (GE Life Sciences, RPN 2235).

### Immunoprecipitation

Protein was extracted with lysis buffer containing 0.1% Triton X-100, supplemented with 7.5 mM MgCl_2_ and lysates were cleared (17,000 × g, 4 °C, 20 minutes). 20 μl of Protein A/G agarose bead slurry (Thermo Scientific, 20421) were added to 25 μl of anti-NLRP3 antibody (CST, 15101) for 30 minutes at 25 °C. Lysates containing 1 mg of total protein were incubated with the bead-antibody conjugate at 4 °C under rotary agitation for 6 hours. Samples were centrifuged (10 sec, 2,000 × g), the supernatant (labelled post-IP) was separated from the beads and kept for validation of successful NLRP3 depletion form the sample. Beads were washed with lysis buffer 5 times, then washed with lysis buffer containing 0.5 M NaCl, then finally washed with normal lysis buffer. The antigen-antibody complex was eluted by heating at 95 °C in SDS-containing loading buffer (Thermo Fisher, NP0007) for 10 minutes. Immunoprecipitation was verified by Western blotting.

### Liquid chromatography-mass spectrometry (LC-MS) for protein sequencing of the immunoprecipitated band by the CST antibody

THP-1 cells were primed with LPS (10 μg/mL) plus ATP (5 mM) for 4 hours and cytosolic protein lysates were prepared as described above. NLRP3 was immunoprecipitated from 7 mg of THP-1 protein lysates as described above using the CST (15101) antibody. Following immunoprecipitation and washes, THP-1 samples were run on a 4–12% Bis-Tris Polyacrylamide gel using pre-stained Molecular weight markers. The desired band (~118 kDa) was excised from the rest of the gel, stored in Milli-Q water and sent to mass spectrometry facility for LC-MS protein sequence analysis with standard procedures of the Harvard core facility.

### Gene expression assays

#### RNA extraction & cDNA synthesis

THP-1 and ARPE-19 cells were lysed in Trizol at 4 °C and passed through a sterile 20-gauge needle. Homogenates were mixed with chloroform (Sigma C2432), samples were centrifuged (12,000 × g, 15 min, 4 °C) and the RNA-containing aqueous phase was transferred to a sterile collection tube. RNA was extracted using a miRNeasy Mini kit (Qiagen, 217004) as per manufacturer’s instructions. RNA purity and concentration were measured using a NanoDrop spectrophotometer; accepted range of RNA purity was 260/280 = 1.9–2.0. 3 μg of RNA were used as a template for cDNA synthesis by means of reverse transcription (RT), as per SuperScript® III kit instructions (Invitrogen, 18080-051) using Oligo(dT)_20_ primer.

#### Quantitative Polymerase Chain Reaction (qPCR)

Relative *NLRP3* mRNA levels were measured from THP-1 and ARPE-19 cells with their accompanying stimuli also tested by Western blotting. TaqMan probes for human *NLRP3* (Hs00918082_m1, Thermo Fisher Scientific, 4331182), mouse *NLRP3* (Mm00840904_m1, Thermo Fisher Scientific, 4331182) and TaqMan Universal PCR Master Mix (Thermo Fisher Scientific, 4304437) were used. *NLRP3* levels were compared to housekeeping genes; mouse 18S rRNA and human *GAPDH*. The amplification protocol (QuantStudio 5 RT-PCR System) consisted of 50 °C for 2 min, 95 °C for 10 min and 40 cycles of 95 °C, 15 seconds and 60 °C, 1 min. Each reaction was performed in triplicates and data was collected from three biologically independent samples (*n* = *3*). For any reaction where no signal was detected at maximum cycles (40), a threshold cycle (C_t_)value of maximum cycles + 1 (41) was manually assigned to the reaction.

### Human donor tissue

Whole human donor globes from AMD patients (*n* = 7 globes, 4 patients 80–94 years old) and age-matched controls (*n* = 6 globes, 4 patients, 76–89 years old) were purchased from the Minnesota Lions Eye Bank (Saint Paul, MN) and the Lions Eye Institute for Transplant & Research (Tampa, FL), in compliance with the Declaration of Helsinki’s principles on human experimentation. Disease severity was assessed using the Minnesota Grading System^56^, eyes were dissected for neuro-retina, macular RPE and peripheral RPE isolation. An incision was performed on the *ora serrata* and the cornea, anterior chamber, lens and vitreous were separated from the posterior chamber, exposing the neuro-retina and pigmented RPE cells. The neuro-retina was peeled off and cut at the optic disc while the RPE were scraped off gently using a transfer pipette. Macular RPE were defined as the cells collected from the area with a diameter equal to twice the distance between the optic disc and the fovea. Macular region surface area was increased to achieve sufficient sample protein concentration. Remaining RPE cells were collected and referred to as peripheral RPE. Protein from retina and RPE was extracted using T-PER (Thermo Fisher, 78510) supplemented with protease inhibitors (Roche, 11836170001), samples were homogenized, sonicated (20% Amp for 10 seconds) and cleared (17,000 × g, 20 minutes, 4 °C) before run on an SDS-gel for Western blotting.

## Electronic supplementary material

Supplementary information
