# Supplementary material for: Issues with the Specificity of Immunological Reagents for NLRP3: Implications for Age-related Macular Degeneration
Source: Sci Rep. 2018 Jan 11;8:461. doi: 10.1038/s41598-017-17634-1 (PMC5764999; doi:10.1038/s41598-017-17634-1)
Supplement: Supplementary file 1 — Supplementary information [file 41598_2017_17634_MOESM1_ESM.pdf]

Supplementary Information

Issues with the Specificity of Immunological Reagents for NLRP3: Implications for AMD Research.

Cassandra Kosmidou[1], Nikolaos E. Efstathiou[1], Mien V. Hoang[1], Shoji Notomi[1], Eleni K. Konstantinou[1], Masayuki Hirano[2], Kosuke Takahashi[2], Daniel E. Maidana[1], Pavlina Tsoka[1], Lucy Young[1], Evangelos S. Gragoudas[1], Timothy W. Olsen[3], Yuki Morizane[2], Joan W. Miller[1] and Demetrios G. Vavvas[1*]

[1]Department of Ophthalmology, Retina Service, Massachusetts Eye and Ear Infirmary, Harvard Medical School, Boston, Massachusetts 02114, USA

[2]Department of Ophthalmology, Okayama University Graduate School of Medicine, Okayama 700-8558, Japan

[3]Department of Ophthalmology, Mayo Clinic, 200 First Street SW, Rochester, MN, 55905, USA

Supplementary Figure S1: NLRP3 Immunoprecipitation validation in THP-1 lysates

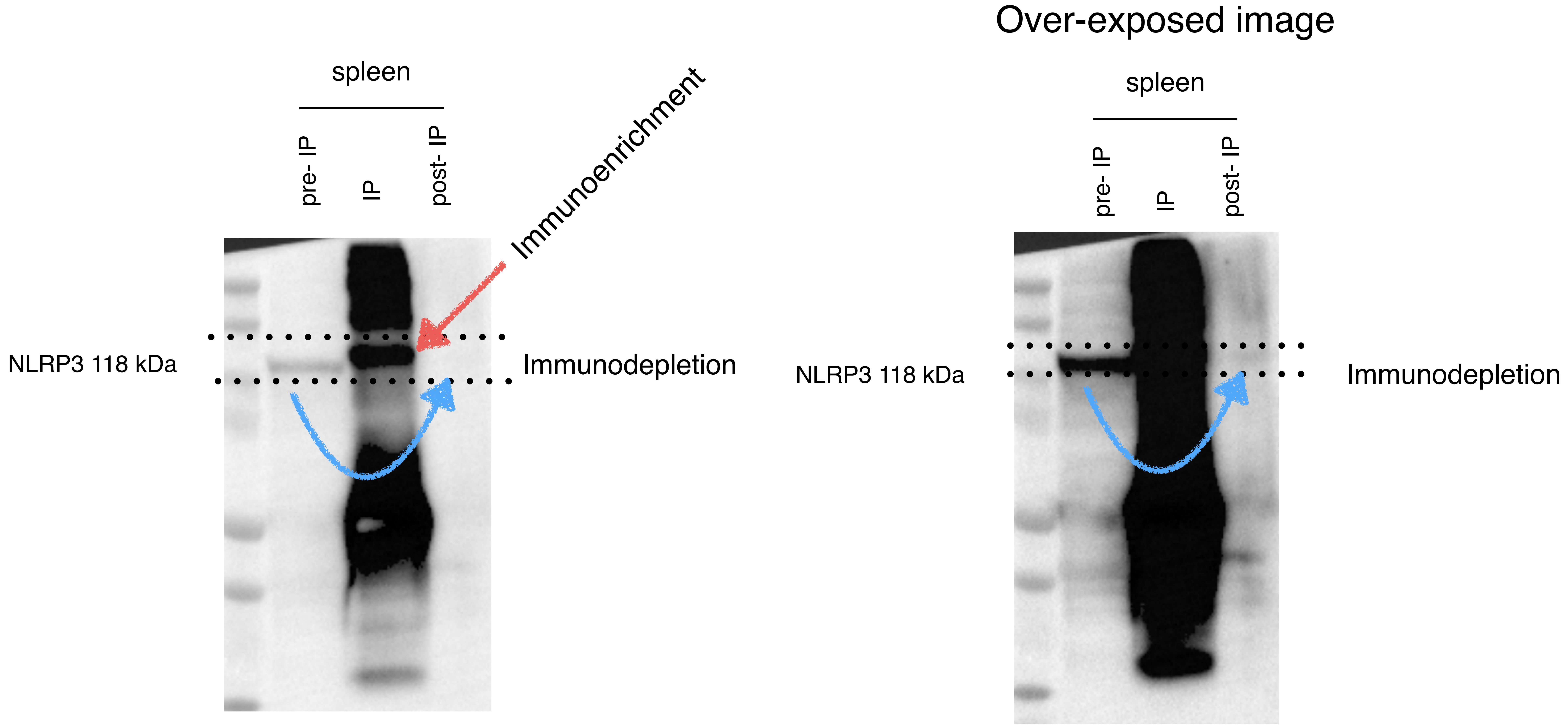

Supplementary Fig. S1: Evidence illustrating that the anti-NLRP3 antibody (CST, D4D8T) can efficiently immunoprecipitate (IP) NLRP3 from positive control THP-1 cells. Western blotting analysis shows a clear band in the pre-IP protein lysate, an enhanced band in the immunoprecipitated (IP) sample (immune-enrichment) and absence of an NLRP3 band in the post-IP protein lysate supernatant, indicating that the sample became depleted of NLRP3 (immune-depletion) and was picked up by the agarose A/G beads—anti-NLRP3 antibody conjugate and collected in the precipitate.

Supplementary Figure S2: Coomassie Blue staining

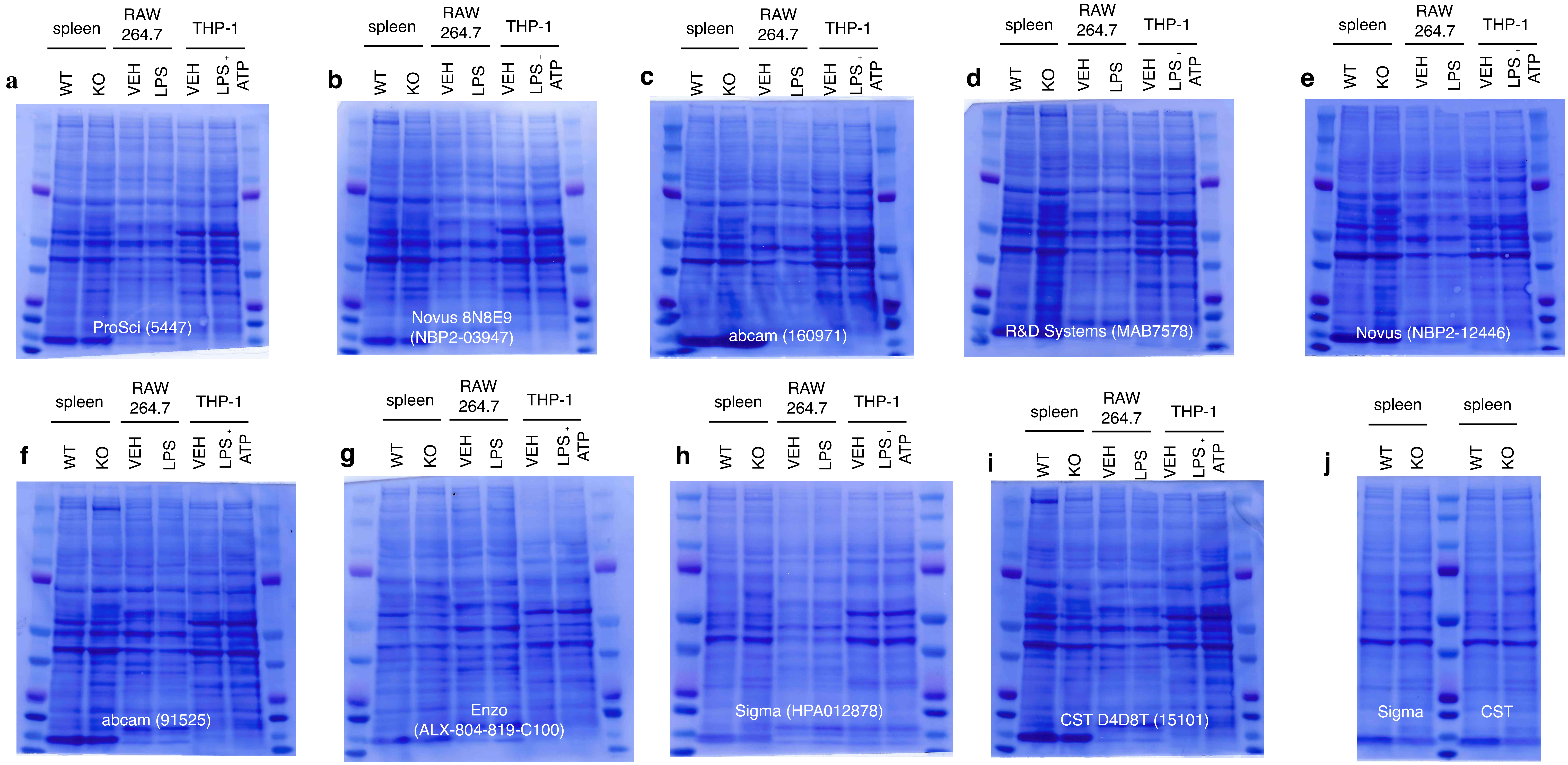

Supplementary Fig. S2: Coomassie Blue staining of the same blots presented in Figures 1a-j, to determine equal protein loading between samples on the same blot.

Supplementary Figure S3: Coomassie Blue staining

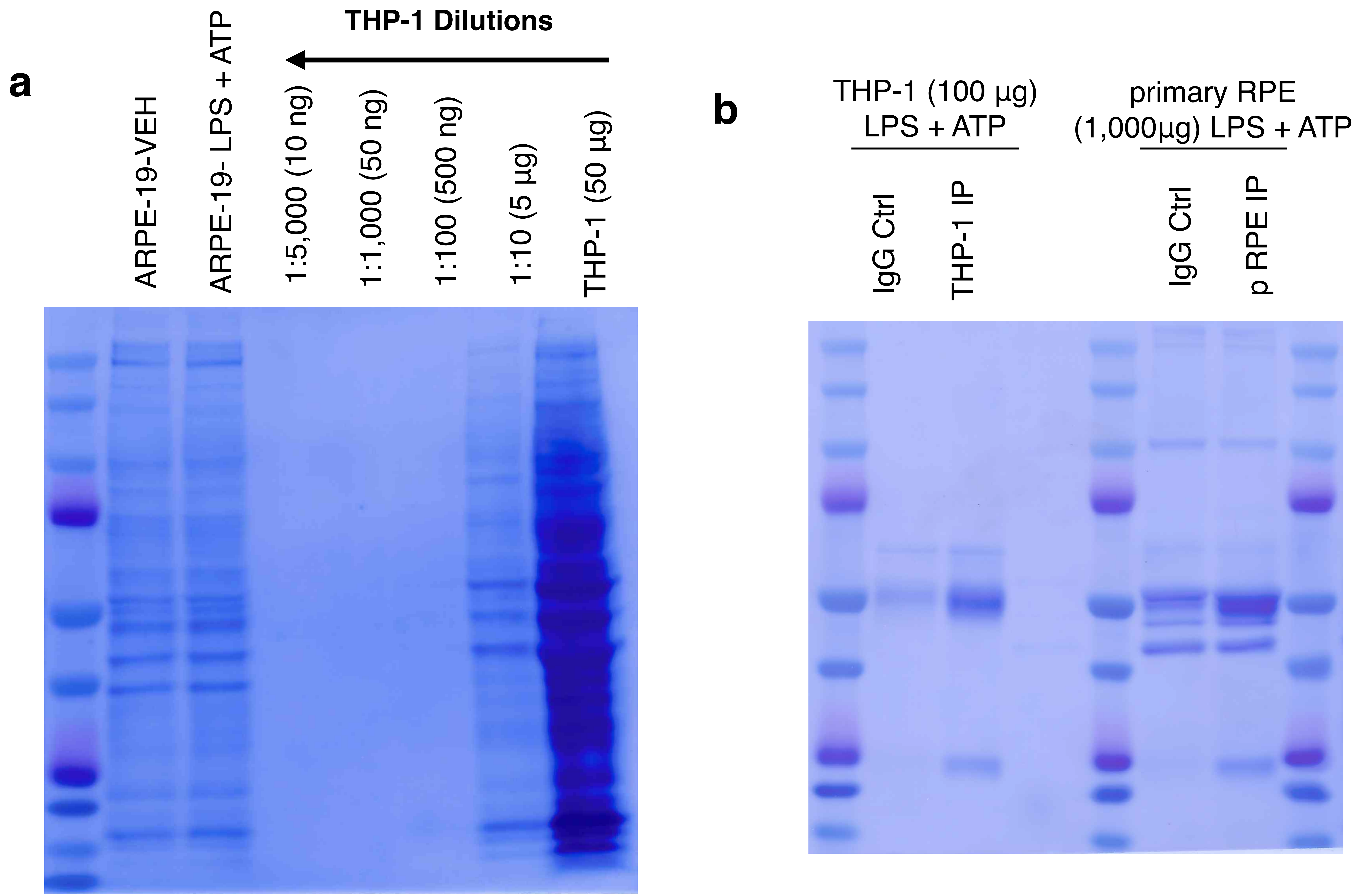

Supplementary Figure S3: Coomassie Blue staining of the same blots presented in Figures 2a, b, to determine equal protein loading between samples on the same blot.

Supplementary Figure S4: Coomassie Blue staining

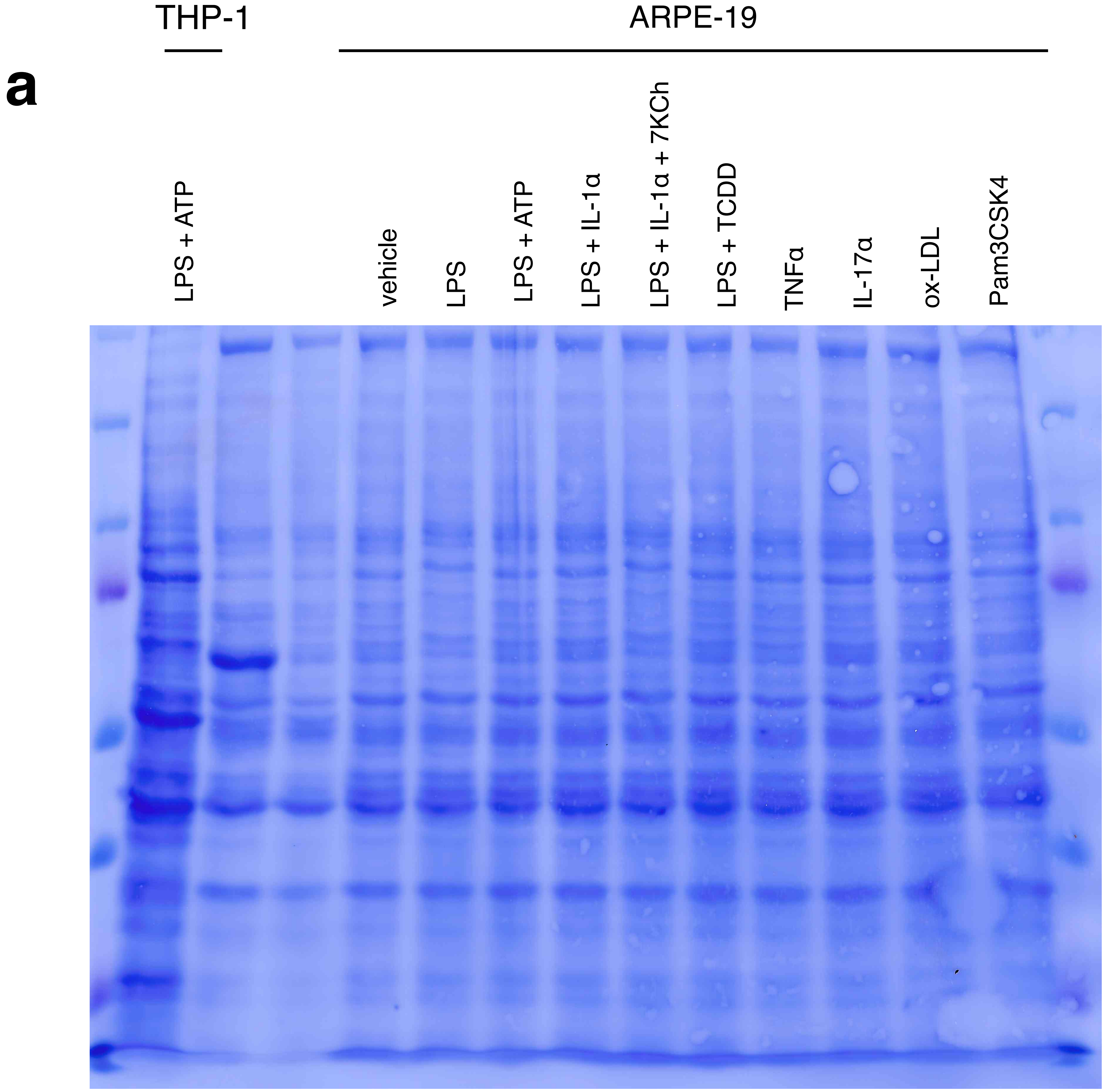

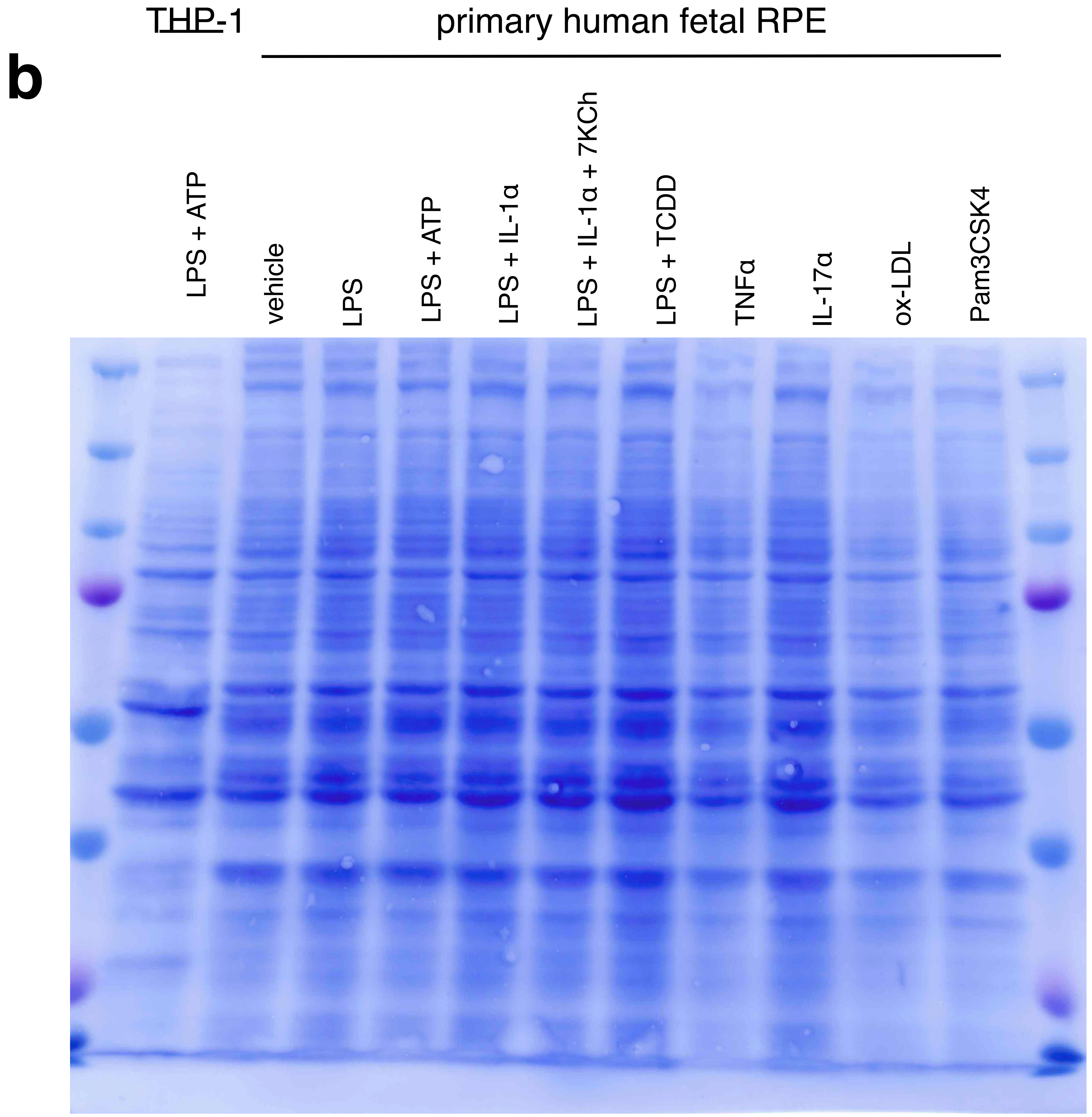

Supplementary Figure S4: Coomassie Blue staining of the same blots presented in Figures 3a, b, to determine equal protein loading between ARPE-19 or primary human fetal RPE samples on the same blot.

Supplementary Figure S5: Coomassie Blue staining

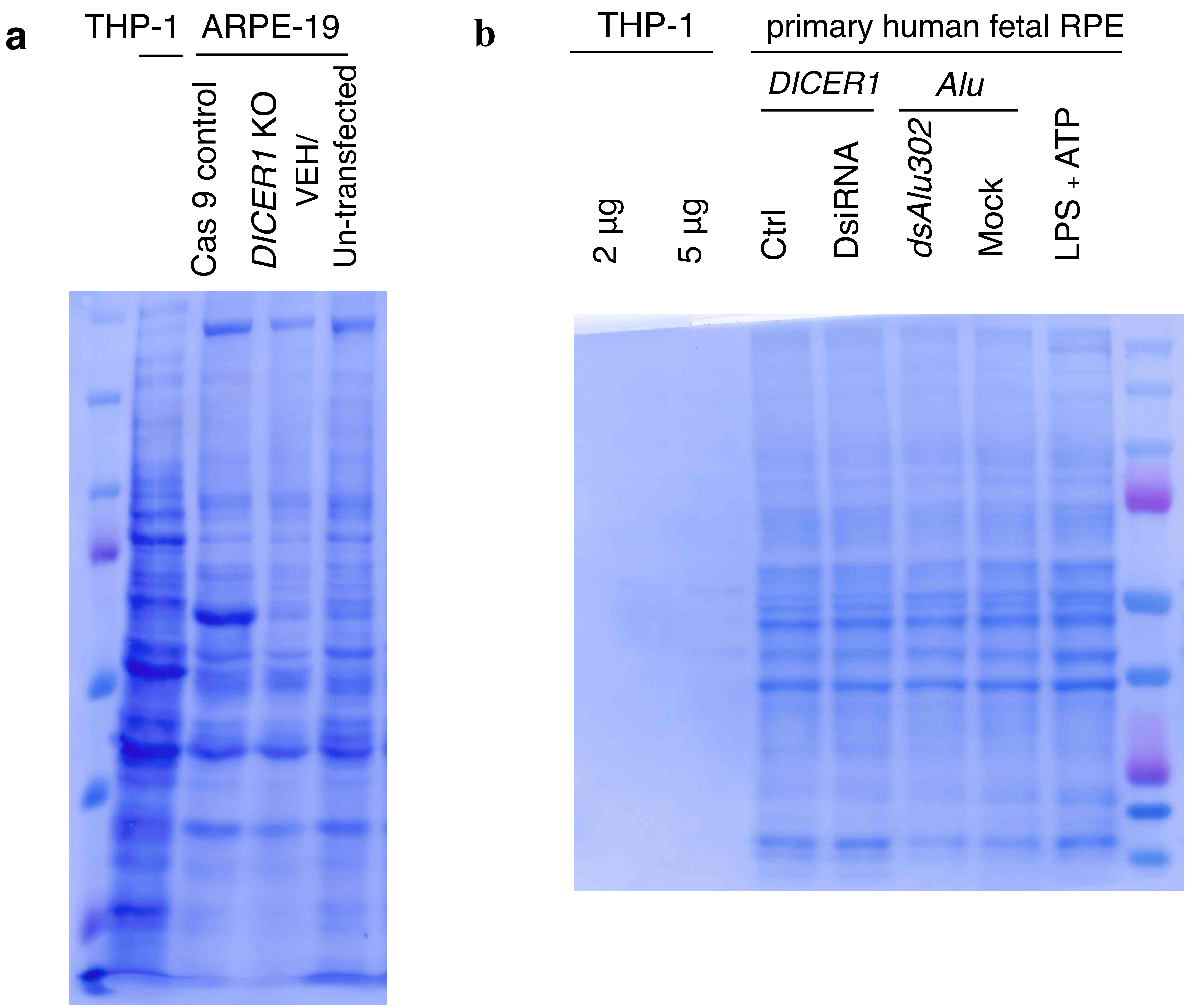

Supplementary Figure S5: Coomassie Blue staining of the same blots presented in Figure 4, to determine equal protein loading between samples on the same blot.

Supplementary Figure S6: Coomassie Blue staining

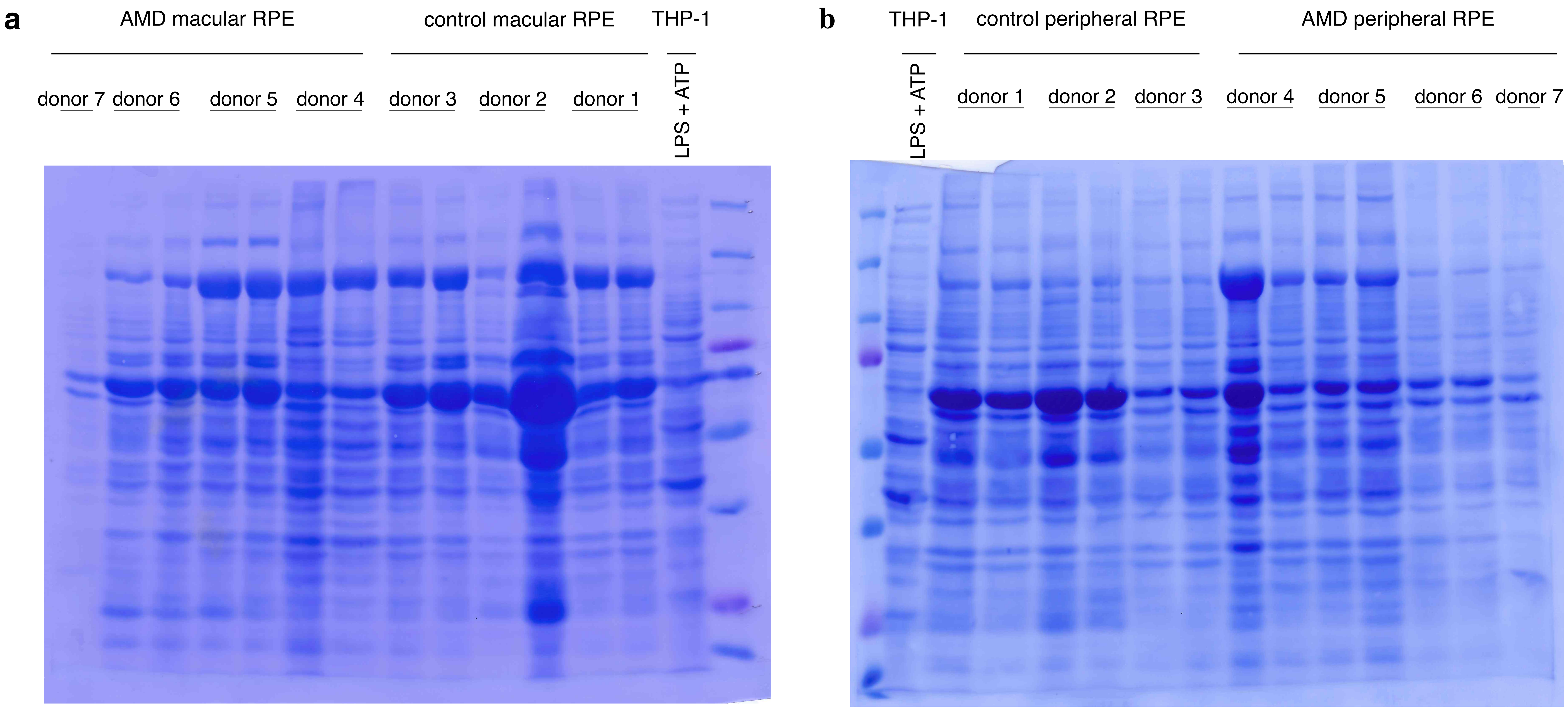

Supplementary Figure S6: Coomassie Blue staining of the same blots presented in Figure 5. To maximize our ability to detect NLRP3 signal we did not perform equal protein loading, which would have limited us to the most dilute sample. Instead maximum amount of protein from each sample was loaded.

Supplementary Figure S7: NLRP3 expression in ARPE-19 and serial dilutions of THP-1

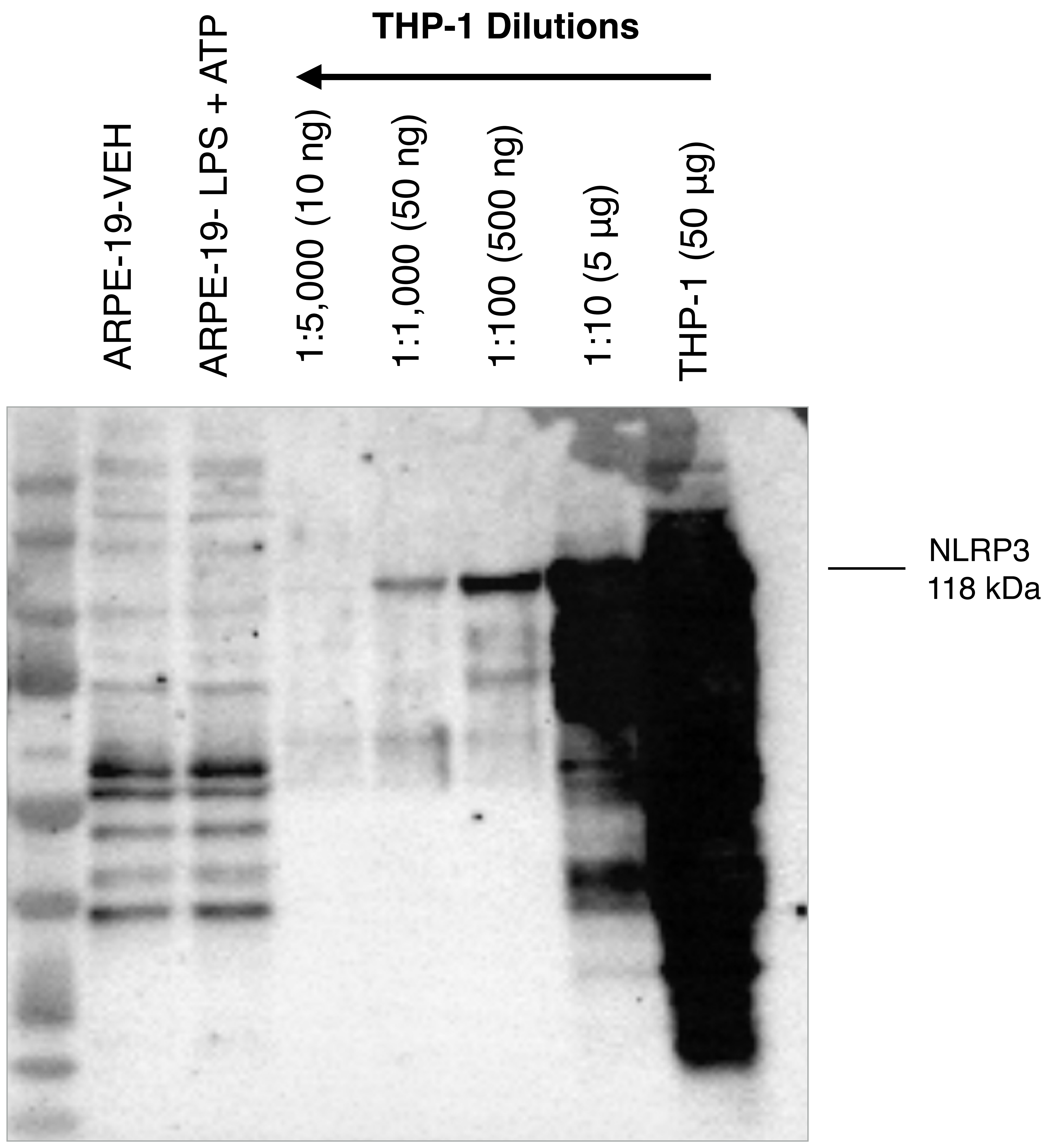

Supplementary Fig. S7 illustrates the whole blot presented at Figure 2a, at a 10-minute exposure time, since in Figure 2a the blot is cropped.

Supplementary Figure S8: Protein sequencing analysis following THP-1 immunoprecipitation

Supplementary Fig. S8 illustrates protein sequencing analysis data: NLRP3 from stimulated THP-1 (LPS + ATP) cells was immunoprecipitated, run on gel electrophoresis and sequenced by mass spectrometry

MATRIX SCIENCE Mascot Search Results

User	: Sunia Trauger
Email	: strauger@fas.harvard.edu
Search title	: sample 1 re-run
MS data file	: C:\Data_MSConvert\Vavaas\sample1_c.mgf
Database	: Human 2013 (88479 sequences; 35079223 residues)
Timestamp	: 3 Nov 2017 at 17:23:01 GMT
Enzyme	: Trypsin
Fixed modifications	: Carbamidomethyl (C)
Mass values	: Monoisotopic
Protein Mass	: Unrestricted
Peptide Mass Tolerance	: ± 5 ppm
Fragment Mass Tolerance	: ± 50 mmu
Max Missed Cleavages	: 1
Instrument type	: ESI-QUAD-TOF
Number of queries	: 10766
Protein hits	: P04264 Keratin, type II cytoskeletal 1 OS=Homo sapiens GN=KRT1 PE=1 SV=6
	Q96P20 NACHT, LRR and PYD domains-containing protein 3 OS=Homo sapiens GN=NLRP3 PE=1 SV=3
	P01857 Ig gamma-1 chain C region OS=Homo sapiens GN=IGHG1 PE=1 SV=1
	K7EQQ3 Keratin, type I cytoskeletal 9 OS=Homo sapiens GN=KRT9 PE=2 SV=1
	Q00839 Heterogeneous nuclear ribonucleoprotein U OS=Homo sapiens GN=HNRNPU PE=1 SV=6

	Human	Decoy	False discovery rate
Peptide matches above identity threshold	6	0	0.00 %
Peptide matches above homology or identity threshold	11	0	0.00 %

Select Summary Report

Format As [Select Summary (protein hits)] Help

Significance threshold p< [0.05] Max. number of hits [AUTO] Show Percolator scores ☐

Standard scoring ◉ MudPIT scoring ○ Ions score or expect cut-off [0] Show sub-sets [1]

Show pop-ups ◉ Suppress pop-ups ○ Require bold red ☐

Preferred taxonomy [All entries]

Re-Search ◉ All queries ○ Unassigned ○ Below homology threshold ○ Below identity threshold

1. P04264 **Mass:** 66170 **Score:** 158 **Matches:** 9(2) **Sequences:** 8(2) **emPAI:** 0.39

Keratin, type II cytoskeletal 1 OS=Homo sapiens GN=KRT1 PE=1 SV=6

Query	Observed	Mr(expt)	Mr(calc)	ppm	Miss	Score	Expect	Rank	Unique	Peptide
9111	487.2677	972.5208	972.5240	-3.21	0	8	3.6	1	U	K.IEISELNR.V
9325	517.2619	1032.5093	1032.5087	0.55	0	14	0.52	1	U	R.TLLEGEESR.M
9684	590.3015	1178.5885	1178.5931	-3.95	0	45	0.00034	1	U	K.YEELQITAGR.H
9897	633.3201	1264.6257	1264.6299	-3.35	0	18	0.19	1	U	R.TNAENEFVTIK.K
9981	650.7670	1299.5195	1299.5224	-2.23	0	5	0.31	1	U	K.NMQDMVEDYR.N
9988	651.8587	1301.7028	1301.7078	-3.83	0	18	0.2	1	U	R.SLDLDSIIAEVK.A
10205	738.3761	1474.7376	1474.7416	-2.69	0	14	0.38	1	U	K.WELLQQVDTSTR.T
10374	858.9276	1715.8406	1715.8438	-1.88	0	36	0.003	1	U	K.QISNLQQSISDAEQR.G 10376

Proteins matching a subset of these peptides:

P04259 **Mass:** 60315 **Score:** 45 **Matches:** 1(1) **Sequences:** 1(1)

Keratin, type II cytoskeletal 6B OS=Homo sapiens GN=KRT6B PE=1 SV=5

P35908 **Mass:** 65678 **Score:** 8 **Matches:** 1(0) **Sequences:** 1(0)

Keratin, type II cytoskeletal 2 epidermal OS=Homo sapiens GN=KRT2 PE=1 SV=2

2. Q96P20 **Mass:** 120662 **Score:** 87 **Matches:** 11(0) **Sequences:** 11(0) **emPAI:** 0.09

NACHT, LRR and PYD domains-containing protein 3 OS=Homo sapiens GN=NLRP3 PE=1 SV=3

Query	Observed	Mr(expt)	Mr(calc)	ppm	Miss	Score	Expect	Rank	Unique	Peptide
8528	439.7396	877.4646	877.4658	-1.34	0	8	2	1	U	K.ADVSAFLR.M
8936	466.2638	930.5130	930.5134	-0.46	0	1	15	1	U	R.EVSLVTQR.S
8999	473.2709	944.5272	944.5291	-2.01	0	14	0.85	1	U	K.IEINLSTR.M
9207	500.7854	999.5562	999.5600	-3.86	0	17	0.17	1	U	R.EQELLAIGK.T
9796	611.8311	1221.6476	1221.6506	-2.49	0	13	0.37	1	U	R.FLFGLVNQER.T
10162	478.5590	1432.6552	1432.6598	-3.17	0	9	0.8	1	U	R.FDYLFYIHCR.E
10353	843.8808	1685.7471	1685.7541	-4.16	0	7	0.64	1	U	K.MMLDWASGTLYQDR.F
10433	923.9810	1845.9474	1845.9472	0.06	0	4	3.2	1	U	K.LVELDLSDNALGDFGIR.L
10488	972.0340	1942.0534	1942.0564	-1.53	0	7	0.98	1	U	K.TTTAVYVFFLSSLLQPR.G
10544	1004.5060	2006.9975	2006.9983	-0.38	0	1	6.7	1	U	R.LYVGENALGDSGVAILCEK.A
10680	1216.6111	2431.2077	2431.2159	-3.35	0	6	1.1	1	U	K.SALETLQEEKPELTVVFEPSW.-

Proteins matching the same set of peptides:

Q96P20-4 **Mass:** 114206 **Score:** 87 **Matches:** 11(0) **Sequences:** 11(0)

Isoform 4 of NACHT, LRR and PYD domains-containing protein 3 OS=Homo sapiens GN=NLRP3

Proteins matching a subset of these peptides:

Q96P20-2 **Mass:** 107958 **Score:** 86 **Matches:** 10(0) **Sequences:** 10(0)

Isoform 1 of NACHT, LRR and PYD domains-containing protein 3 OS=Homo sapiens GN=NLRP3

Q96P20-5 **Mass:** 114414 **Score:** 86 **Matches:** 10(0) **Sequences:** 10(0)

Isoform 5 of NACHT, LRR and PYD domains-containing protein 3 OS=Homo sapiens GN=NLRP3

Q96P20-3 **Mass:** 84733 **Score:** 76 **Matches:** 8(0) **Sequences:** 8(0)

Isoform 3 of NACHT, LRR and PYD domains-containing protein 3 OS=Homo sapiens GN=NLRP3

3. P01857 **Mass:** 36596 **Score:** 31 **Matches:** 2(1) **Sequences:** 2(1) **emPAI:** 0.10

Ig gamma-1 chain C region OS=Homo sapiens GN=IGHG1 PE=1 SV=1

Query	Observed	Mr(expt)	Mr(calc)	ppm	Miss	Score	Expect	Rank	Unique	Peptide
8218	418.2194	834.4243	834.4269	-3.09	0	28	0.03	1	U	K.DTLMISR.T
8243	419.7549	837.4953	837.4960	-0.83	0	3	2.4	1	U	K.ALPAPIEK.T

Proteins matching the same set of peptides:

P01860 **Mass:** 42287 **Score:** 31 **Matches:** 2(1) **Sequences:** 2(1)

Ig gamma-3 chain C region OS=Homo sapiens GN=IGHG3 PE=1 SV=2

Proteins matching a subset of these peptides:

P01859 **Score:** 28 **Matches:** 1(1) **Sequences:** 1(1)

Ig gamma-2 chain C region OS=Homo sapiens GN=IGHG2 PE=1 SV=2

P01861 **Score:** 28 **Matches:** 1(1) **Sequences:** 1(1)

Ig gamma-4 chain C region OS=Homo sapiens GN=IGHG4 PE=1 SV=1

4. K7EQQ3 **Mass:** 39618 **Score:** 28 **Matches:** 1(1) **Sequences:** 1(1) **emPAI:** 0.09

Keratin, type I cytoskeletal 9 OS=Homo sapiens GN=KRT9 PE=2 SV=1

Query	Observed	Mr(expt)	Mr(calc)	ppm	Miss	Score	Expect	Rank	Unique	Peptide
9631	579.2972	1156.5798	1156.5836	-3.32	0	28	0.015	1	U	R.QGVDADINGLR.Q

Proteins matching the same set of peptides:

P35527 **Mass:** 62255 **Score:** 28 **Matches:** 1(1) **Sequences:** 1(1)

Keratin, type I cytoskeletal 9 OS=Homo sapiens GN=KRT9 PE=1 SV=3

5. Q00839 **Mass:** 91269 **Score:** 27 **Matches:** 1(1) **Sequences:** 1(1) **emPAI:** 0.04

Heterogeneous nuclear ribonucleoprotein U OS=Homo sapiens GN=HNRNPU PE=1 SV=6

Query	Observed	Mr(expt)	Mr(calc)	ppm	Miss	Score	Expect	Rank	Unique	Peptide
10373	857.9597	1713.9049	1713.9050	-0.07	0	27	0.016	1	U	K.SSGPTSLFAVTVAPPGAR.Q

Proteins matching the same set of peptides:

Q00839-2 **Mass:** 89665 **Score:** 27 **Matches:** 1(1) **Sequences:** 1(1)

Isoform Short of Heterogeneous nuclear ribonucleoprotein U OS=Homo sapiens GN=HNRNPU

Mascot: http://www.matrixscience.com/

Supplementary Table S1: Summary of immunological reagents used in the present study.

WB: Western Blot, IP: Immunoprecipitation

	Manufacturer	Reactivity	Host	[antibody]	Catalogue #	Lot #
anti-NLRP3	Cell Signaling Technologies	mouse, human	rabbit	WB 1:1000	D4D8T, 15101	3
	ProSci	mouse, human	rabbit	WB 1:1000	5447	6769-1204
	Novus Biologicals	mouse, human	mouse	WB 1:1000	8N8E9, NBP2-03947	-
	Novus Biologicals	mouse, human	rabbit	WB 1: 400	NBP2-12446	080639650-14
	abcam	human	mouse	WB 1:1000	ab160971	GR228391-15
	R&D Systems	mouse	rat	WB 1: 250	MAB7578	-
	abcam	human, mouse	rabbit	WB 1: 500	ab91525	GR62279-6
	Enzo Life Sciences	human	mouse	WB 1:100	ALX-804-818-C100	11051424
	Sigma	human	rabbit	WB 1:1000	HPA012878	D106015
anti-DICER1	Cell Signaling Technologies	human	rabbit	WB 1:1000	3363	-
anti-ds RNA	SCICIONS	50 species	mouse	IF 1: 250	10010200	J2-1611
anti-Rabbit IgG	Sigma	rabbit	mouse	IP 0.2 ng/ml	R2655	-

Supplementary Table S2: guide RNA sequences for the establishment of *DICER1* knockout ARPE-19 cells

	Sequence	Score	Targeted exon
**Guide #1**	5'- CACCGCATGTGGAATCGCTTATATC-3’	89%	1
**Guide #2**	5’- CACCGCTTCCATCTGTTTGTTGCG -3’	82%	2
**Guide #3**	5’- CACCGTACTGATATAAGCCAGCTC - 3’	75%	2
